# Synergistic innate-adaptive immunity by NKG2D-specific CAR-macrophages drives durable remission in hepatocellular carcinoma

**DOI:** 10.1186/s12943-025-02538-w

**Published:** 2025-12-13

**Authors:** Zihao Zhao, Wenjing Zheng, Yang He, Han Zhang, Lingling Zhang, Yi Huo, Junwei Jiang, Chen Zhang, Haohan Lyu, Weiwei Qin, Chen Liu, Feng Chang, Lequn Shan, Tao Wang, Wenjie Song

**Affiliations:** 1 Department of Hepatobiliary Surgery, Xijing Hospital, Fourth Military Medical University, Xi’an, China; 2 State Key Laboratory of Holistic Integrative Management of Gastrointestinal Cancers, Department of Medical Genetics and Developmental Biology, Fourth Military Medical University, Xi’an, China; 3 Department of Urology, Xijing Hospital, Fourth Military Medical University, Xi’an, China; 4 Department of Spine Surgery, Honghui Hospital, Xi’an Jiaotong University, Xi’an, China; 5 Department of Hematology, Tangdu Hospital, Fourth Military Medical University, Xi’an, China

**Keywords:** Chimeric antigen receptor macrophages, NKG2D ligands, Phagocytosis, Hepatocellular carcinoma, Tumor microenvironment

## Abstract

**Background:**

Hepatocellular carcinoma (HCC) immunotherapy is limited by antigenic heterogeneity and an immunosuppressive microenvironment. This study engineered chimeric antigen receptor macrophages (CAR-Ms) targeting stress-inducible NKG2D ligands (NKG2DLs), broadly overexpressed in HCC, to enhance phagocytic clearance and remodel immunity.

**Methods:**

NKG2DL expression in HCC and association with survival were analyzed. CAR-Ms were constructed by fusing the NKG2D extracellular domain to FcγRI signaling. In vitro assays assessed phagocytosis, cytokine secretion, signaling, and T cell interactions. Therapeutic efficacy was evaluated in immunocompetent mice bearing subcutaneous, orthotopic, or metastatic HCC models, with or without anti-PD-L1. Tumor progression, immunity, and survival were analyzed via bioluminescence imaging, flow cytometry, histopathology, and serum biochemistry. Statistics analyses were performed using t-tests, ANOVA, and log-rank tests.

**Results:**

NKG2DLs were significantly upregulated in human HCC and correlated with poor prognosis. CAR-Ms selectively engulfed NKG2DL⁺ tumor cells, polarized to an M1 phenotype, and activated PI3K-AKT and cGAS-STING pathways, driving phagocytosis and pro-inflammatory cytokines secretion. They enhanced T cell chemokines (Cxcl10, Ccl5) and antigen presentation, boosting T cell recruitment and activation in vitro. In subcutaneous models, CAR-Ms suppressed tumor growth, reprogrammed tumor-associated myeloid cells toward M1, and induced durable immune memory (100% tumor rejection upon rechallenge), with T cell activation. In orthotopic models, CAR-M monotherapy induced complete regression by week 5 and 100% survival, with elevated CD8⁺ T cells and CAR-M specifically homing to liver tumors. CAR-Ms suppressed metastasis in peritoneal/pulmonary models. Combining CAR-Ms with PD-L1 blockade accelerated tumor clearance and survival versus monotherapies, enhancing T cell cytotoxicity. Safety assessments showed no significant organ toxicity based on histopathology and serum biochemistry.

**Conclusions:**

NKG2D-directed CAR-Ms eliminate HCC through integrated innate phagocytosis, adaptive immune activation, and myeloid reprogramming, overcoming key therapeutic barriers. Combination with anti-PD-L1 enhances therapeutic efficacy by leveraging innate-adaptive crosstalk, providing a promising approach for HCC immunotherapy.

**Supplementary Information:**

The online version contains supplementary material available at 10.1186/s12943-025-02538-w.

## Background

Hepatocellular carcinoma (HCC) ranks as the sixth most common malignancy worldwide and the third leading cause of cancer-related deaths, with a persistently poor 5-year survival rate of 18% [1, 2]. Although surgical and locoregional therapies (e.g., resection or ablation) have advanced, over 50% of patients still require systemic treatments [3, 4], and even curative interventions for early-stage HCC face recurrence rates exceeding 70% [5]. Immune checkpoint inhibitors targeting the PD-1/PD-L1 axis now serve as first-line immunotherapies, yet their clinical efficacy remains limited, with objective response rates of only 15–30% [6, 7]. This suboptimal performance is attributed to HCC’s immunosuppressive tumor microenvironment (TME), characterized by antigenic heterogeneity, T cell exhaustion, and myeloid cell-driven immunosuppression (e.g., M2-polarized macrophages and regulatory T cells) [7]. Moreover, the lack of durable tumor control in responders underscores the urgent need for strategies that not only reinvigorate adaptive immunity but also directly remodel the immunosuppressive TME.

While adoptive cell therapies hold promise for HCC, conventional approaches such as CAR-T cells face critical limitations in solid tumors. These include inadequate infiltration into dense stromal regions and suppression by immunosuppressive myeloid populations (e.g., M2-polarized macrophages) that dominate the HCC microenvironment [8–10]. In contrast, macrophages possess unique biological properties ideally suited for solid tumor therapy. Their inherent capacity to home to hypoxic tumor niches, penetrate fibrotic stroma, and dynamically adapt to microenvironmental cues positions them as potent effector cells [11]. Pioneering work by Klichinsky et al. demonstrated that CAR-macrophages (CAR-M) not only directly engulf antigen-positive tumor cells but also remodel the immunosuppressive TME via pro-inflammatory cytokine secretion (e.g., TNF-α, IL-12) and recruitment of cytotoxic T cells [12]. Unlike CAR-T cells, which primarily rely on cytotoxic granule release, CAR-Ms execute dual antitumor mechanisms: (1) innate phagocytic clearance of tumor cells and (2) adaptive immune activation through antigen cross-presentation and chemokine-mediated T cell recruitment. This multifunctionality allows CAR-Ms to simultaneously target tumor cells and disrupt immunosuppressive barriers, offering a distinct therapeutic advantage through their dual mechanism that current lymphocyte-based approaches cannot fully replicate [13–15].

Target selection is pivotal for CAR-M efficacy in HCC, where antigenic heterogeneity and immunosuppression pose formidable barriers. The NKG2D receptor-ligand axis emerges as a compelling therapeutic target due to its intrinsic role in tumor immunosurveillance. NKG2D ligands (NKG2DLs), including MICA/B and ULBP1-6, are stress-inducible molecules selectively upregulated on malignant cells during oncogenic stress or genomic instability, while remaining minimally expressed in healthy tissues [16]. This tumor-restricted expression profile minimizes off-tumor toxicity risks—a critical consideration for solid tumor therapies. Unlike conventional CAR designs dependent on synthetic single-chain variable fragments (scFvs), NKG2D-based CARs leverage the endogenous receptor’s natural architecture, thereby reducing immunogenicity and enhancing structural stability. Furthermore, the polyvalent ligand-binding capacity of NKG2D enables recognition of multiple stress-induced ligands (e.g., MICA, ULBP2), effectively circumventing antigen escape—a major limitation of single-antigen-targeted therapies in solid tumors. These intrinsic advantages position the NKG2D-NKG2DL axis as a strategically superior target for CAR-M therapy in HCC.

In this study, we engineered a novel CAR-M platform by fusing the extracellular domain of NKG2D with the FcγRI signaling module. The engineered CAR-Ms exhibited FcγRI-dependent phagocytosis and M1 polarization, driven by coordinated activation of the PI3K-AKT and cGAS-STING pathways, which are central to innate immune activation. Beyond direct tumor clearance, CAR-Ms secreted T cell-recruiting chemokines (e.g., Cxcl10), facilitated antigen presentation to amplify T cell priming, and reprogrammed immunosuppressive myeloid populations toward an immunostimulatory M1 phenotype. The in vivo distribution of CAR-Ms was organ-specific, with preferential accumulation in liver tumor sites, which may contribute to their efficacy in orthotopic liver cancer models. These multifunctional capabilities collectively drove robust antitumor activity across preclinical models. Notably, combination therapy with anti-PD-L1 enhanced both CAR-M-mediated phagocytosis and T cell cytotoxicity, resulting in accelerated tumor eradication and improved survival outcomes compared to monotherapies. This strategy—simultaneously targeting innate phagocytosis, adaptive immunity, and myeloid reprogramming—addresses the dual barriers of antigenic heterogeneity and immunosuppression, providing a promising approach for HCC immunotherapy.

## Methods

### Cell lines and culture conditions

Human HEK-293T cells and murine iBMDM, GL261, B16F10, H22, Hepa1-6, 4T1, CT26, ID8 and MC38 cell lines were purchased from Procell Life Science & Technology (Wuhan, China). Cells were cultured in RPMI-1640 (Gibco, 11875-085) or DMEM (Gibco, 10569010) supplemented with 10% fetal bovine serum (ExCell, FSS500) and 1% penicillin-streptomycin (Gibco, 15140-122). All cultures were maintained at 37 °C in a 5% CO₂ incubator.

For in vitro and in vivo experimental tracking, tumor cell lines were infected by lentivirus encoding luciferase2, mScarlet, and puro (pLenti-luciferase2-mScarlet-Puro) to generate stable bioluminescent reporter cell lines by puromycin selection. Additionally, H22 and B16F10 cells were infected with pLenti-tRae-1β-mScarlet-puro lentivirus, which encodes the extracellular and transmembrane domains of Rae-1β without the intracellular signaling domain. Stable clones expressing truncated Rae-1β were established through puromycin selection.

### Plasmid construction and lentivirus production

CAR constructs were cloned into a third-generation lentiviral backbone using standard molecular biology techniques. Briefly, constructs comprising the CD8 signal peptide, NKG2D extracellular domain, CD28 hinge region, and FcγRI transmembrane/intracellular domains were driven by the SFFV promoter. All cloning steps were validated by restriction enzyme digestion and sequencing. GFP was included as a reporter gene. Lentiviral particles were packaged in HEK293T cells and harvested following established protocols for ultracentrifugation-based purification and concentration.

### Flow cytometry analysis

CAR expression on iBMDM-derived macrophages was assessed via two-step staining with mouse anti-CD314 (NKG2D)- phycoerythrin (PE) (BioLegend, 130207). NKG2D ligand (NKG2DL) subtypes were detected using: anti-mH60 (R&D Systems, MAB1155), anti-mRae-1 (R&D Systems, MAB17582), anti-mMult-1 (R&D Systems, MAB2588), followed by goat anti-rat IgG-FITC (BioLegend, 405404). Macrophage polarization markers (M1/M2) were analyzed with: anti-CD11b-PE/Cyanine7 (BioLegend, 101216), anti-CD80-PerCP/Cyanine5.5 (BioLegend, 104721), anti-CD86-APC/Cyanine7 (BioLegend, 105029), anti-CD206-PE (Invitrogen, 12–2061-80), anti-CD163-APC (Invitrogen, 17–1631-82), and annexin V-PE (BD Biosciences, 559763) for apoptosis detection. Murine tumor-infiltrating immune cells were characterized using: anti-CD45-BV510 (BioLegend, 103138), anti-CD3-APC (BioLegend, 100236), anti-CD4-PE (BioLegend, 100407), anti-CD8a-FITC (BioLegend, 100706), and anti-B220-PerCP (BioLegend, 103233). T cell activation/exhaustion markers included anti-CD69-PE (BioLegend, 104507), anti-IFN-γ-APC/Cyanine7 (BioLegend, 505849), anti-TNFα-PE (Invitrogen, 12–7321-81), anti-PD-1-PE (Invitrogen, 12–9985-81), and anti-PD-L1-PE (Invitrogen, 12–5983-41). Isotype controls matched to fluorophore profiles were obtained from BioLegend. Flow cytometry data were acquired on BD FACS Canto™ II (BD Biosciences) or CytoFLEX (Beckman Coulter) and analyzed with FlowJo (v10.8.1) or CytExpert (v2.4).

### FACS-based phagocytosis assay

To quantify phagocytic activity, 5 × 10⁵ GFP-M or CAR-M were co-cultured with 5 × 10⁵ target cells (B16F10, Hepa1-6, MC38, B16F10-Rae-1β, H22, or H22-Rae-1β) stably expressing mScarlet in DMEM or RPMI-1640 supplemented with 10% FBS at 37 °C for 1 h. Following incubation, cells were harvested, washed twice with cold PBS, and resuspended in FACS buffer (PBS + 2% FBS). Flow cytometry was performed using BD FACS Canto™ II (BD Biosciences) to identify double-positive (GFP⁺PE⁺) events, where GFP marks macrophages and the PE signal identifies engulfed tumor cell debris. Phagocytosis efficiency was calculated as the percentage of GFP⁺ cells co-expressing PE signal. Data were analyzed with FlowJo v10.8.1, with triplicate technical replicates per condition.

###  In vitro cytotoxicity assay

Tumor cell lysis was assessed via luciferase-based cytotoxicity assays. Hepa1-6 and MC38 cells stably expressing firefly luciferase (Luc2-mScarlet-Puro) served as targets. CAR-M or GFP-M were seeded in 96-well plates at varying effector-to-target (E: T) ratios (1:1, 3:1, 5:1, 10:1). After 6-hour pre-incubation, tumor cells were added, and co-cultures were maintained for 24–48 h at 37 °C. In certain experiments, 1 µM BKM120 (Selleck, S2247) or 0.5 µM H-151(SparkJade, SJ-MX0230) were added to the culture medium to block PI3K or STING, respectively. Luciferase activity was quantified using an IVIS Spectrum imaging system (PerkinElmer) following D-luciferin (Life-iLab, AC19L014) substrate addition (150 µg/mL final concentration). Bioluminescence signals were captured with a 1-minute exposure time. Cell lysis was calculated as:

$$\begin{aligned}\mathrm{Lysis}\;(\%) = &(\mathrm{Maximum}\;\mathrm{Lysis}\;\mathrm{Signal} - \mathrm{Tumor\mbox{-}Only}\;\mathrm{Signal}) \\& / (\mathrm{Sample}\;\mathrm{Signal} - \mathrm{Tumor\mbox{-}Only}\;\mathrm{Signal}) \times 100\end{aligned}$$

Maximum lysis was defined by tumor cells lysed with 1% Triton X-100. Experiments were repeated thrice independently, with six technical replicates per group.

### Live-cell high-resolution time-lapse imaging

To visualize dynamic interactions between CAR-M and tumor cells, 5 × 10⁵ GFP-M or CAR-M were co-cultured with 5 × 10⁵ Luc2-mScarlet-Puro Hepa1-6 cells in glass-bottom dishes (MatTek, P35G-1.5–14.5-C). Live-cell imaging was performed on an IXplore SpinSR confocal microscope (Olympus) equipped with a 20× air objective (NA 0.75) and environmental chamber (37 °C, 5% CO₂). Fluorescence excitation/emission wavelengths were set to 488 nm/500–550 nm (GFP) and 561 nm/570–620 nm (mScarlet). Time-lapse sequences were acquired at 5-minute intervals over 2 h using Olympus IXplore software. Image stacks were processed with Fiji (v2.3.0) for background subtraction and contrast enhancement. Representative videos were compiled at 10 frames/second to capture phagocytic events and macrophage motility.

### Quantitative Real-Time PCR (qRT-PCR)

Total RNA was extracted from cells using the SPARKeasy RNA Extraction Kit (SparkJade, AC0205) and quantified via NanoDrop ND-1000 (Thermo Fisher Scientific). Complementary DNA (cDNA) was synthesized from 1 µg RNA using PrimeScript II Reverse Transcriptase (Takara, 2690 A) with random hexamer primers. qRT-PCR was performed on a CFX Connect Real-Time System (Bio-Rad) with TB Green^®^ Fast qPCR Mix (Takara, RR430A) according to the manufacturer’s protocol. Primers (sequences below) were designed to span exon-exon junctions for specificity: Mouse *IL-6* : Forward: 5′- CCACTTCACAAGTCGGAGGCTTA − 3′, Reverse: 5′- GCAAGTGCATCATCGTTGTTCATAC − 3′; Mouse *IL-10* : Forward: 5′- CCCTTTGCTATGGTGTCCTT − 3′, Reverse: 5′- TGGTTTCTCTTCCCAAGACC − 3′; Mouse *IFN-γ*: Forward: 5′- GAACTGGCAAAAGGATGGTGA − 3′, Reverse: 5′- TGTGGGTTGTTGACCTCAAAC − 3′; Mouse *CD80* : Forward: 5′-TCAGTTGATGCAGGATACACCA-3′, Reverse: 5′-AAAGACGAATCAGCAGCACAA-3′;Mouse *CD86* :Forward: 5′-TCAATGGGACTGCATATCTGCC-3′, Reverse: 5′-GCCAAAATACTACCAGCTCACT-3′; Mouse *TNF-α* :Forward: 5′-ACGGCATGGATCTCAAAGAC-3′, Reverse: 5′-AGATAGCAAATCGGCTGACG-3′; Mouse *IL-12p35* : Forward: 5′-AATGTCTGCGTGGAAGCTCA-3′, Reverse: 5′-ATGCCCACTTGCTGCATGA-3′; Mouse *IL-1β* : Forward: 5′-GCAACTGTTCCTGAACTCAACT-3′, Reverse: 5′-ATCTTTTGGGGTCCGTCAACT-3′; Mouse Gapdh (internal control) : Forward: 5′-AGGTCGGTGTGAACGGATTTG-3′, Reverse: 5′-GGGGTCGTTGATGGCAACA-3′. Relative gene expression was calculated using the ΔΔCt method, with *Gapdh* as the reference gene. Each sample was analyzed in triplicate, and experiments were repeated independently three times to ensure reproducibility.

### Statistical analysis

Data analysis was performed using R (v4.3.1) and GraphPad Prism 9.0. Results are presented as mean ± standard deviation (SD). For comparisons between two groups, unpaired Student’s *t* -test was applied. One-way analysis of variance (ANOVA) with Tukey’s post hoc test was used for multiple-group comparisons. Survival curves were analyzed by Kaplan-Meier method with log-rank test. Statistical significance was defined as *p* < 0.05: *p < 0.05, **p < 0.01, ***p < 0.001. All experiments were independently repeated at least three times to ensure reproducibility.

Additional methodological details are described in the Supplementary Materials.

## Results

### NKG2DLs overexpression correlates with aggressive HCC phenotype and guides CAR-M development

**Fig. 1:**
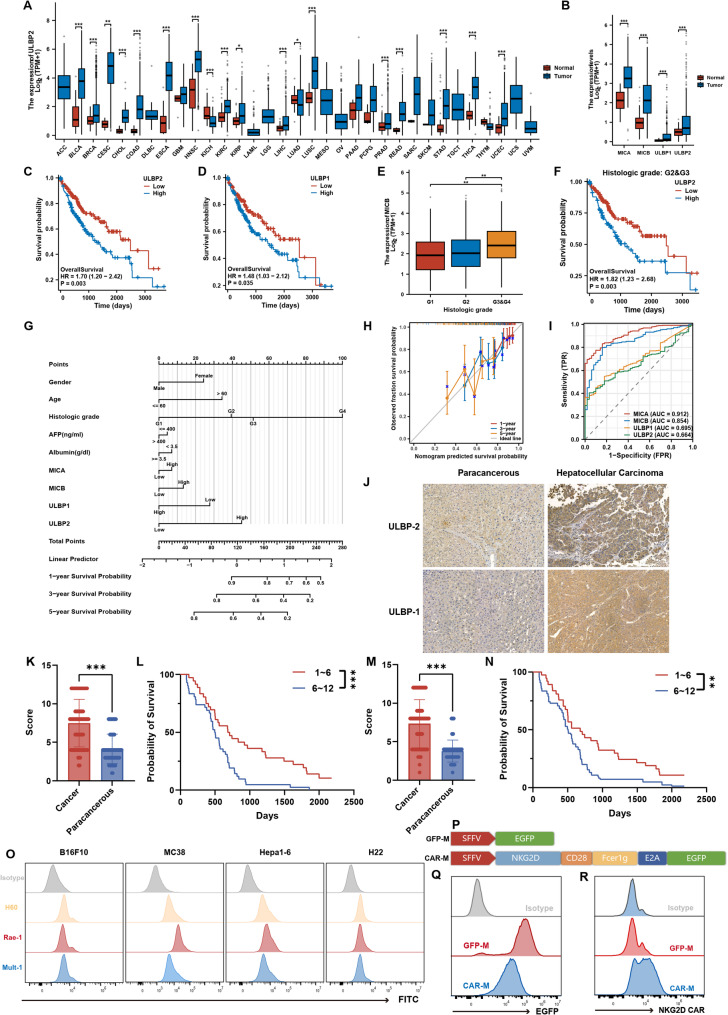
NKG2DLs are overexpressed in hepatocellular carcinoma and correlate with poor prognosis. **A** Pan-cancer analysis of NKG2DL expression profiles across multiple tumor types from the TCGA database. **B** Differential expression of major NKG2DL subtypes between human HCC and matched adjacent non-tumorous liver tissues. **C**-**D** Kaplan-Meier overall survival curves of HCC patients stratified by high vs. low expression of ULBP2 (**C**) and ULBP1 (**D**). **E** NKG2DL expression levels across different HCC histological grades. **F** Survival analysis based on NKG2DL expression within distinct histological grade subgroups. **G** A nomogram integrating NKG2DL expression and clinical parameters for predicting 1-, 3-, and 5-year overall survival. **H** Calibration curves assessing the concordance between nomogram-predicted and observed survival probabilities. **I** Time-dependent receiver operating characteristic (ROC) analysis evaluating the diagnostic performance of NKG2DLs for predicting patient survival. **J** Representative IHC images of ULBP2 and ULBP1 in human HCC and adjacent non-tumorous tissues. **K**–**N** Quantitative IHC scores for ULBP2 (**K**) and ULBP1 (**M**) in paired tumor and non-tumor specimens, with their corresponding Kaplan-Meier survival analyses (**L**, **N**). **O** Flow cytometry analysis of NKG2DL surface expression on a panel of murine tumor cell lines. **P** Schematic diagram of the lentiviral construct designs for GFP-M (control) and NKG2D-targeting CAR-M. **Q** Efficiency of lentiviral transduction in iBMDM-derived macrophages, as indicated by EGFP reporter expression. **R** Surface expression levels of the engineered NKG2D-CAR on macrophages, confirmed by flow cytometry

The NKG2D-NKG2DL axis represents a fundamental immunosurveillance mechanism through ligand-receptor interaction. Analysis of TCGA datasets revealed pan-cancer overexpression of NKG2DLs (Fig. 1A, Fig. S1A), with HCC tissues showing significantly elevated NKG2DL levels compared to adjacent non-tumorous tissues (Fig. 1B). Clinopathological correlations demonstrated that high NKG2DL expression associates with shortened patient survival (Fig. 1C-D, Fig. S1B), advanced histological grades (Fig. 1E, F, Fig. S1D, E), and serves as an independent prognostic factor in multivariate analysis (Fig. 1G). A composite nomogram integrating NKG2DL expression and clinical parameters exhibited strong predictive accuracy, as evidenced by calibration curves aligning predicted and observed survival outcomes (Fig. 1H). Diagnostic ROC analysis identified MICA/MICB as robust discriminators of HCC progression (Fig. 1I, Fig. S1C). Immunohistochemical validation confirmed upregulated ULBP1/2 expression in HCC tissues compared to normal counterparts (Fig. 1J, K, M), with higher expression correlating to poor differentiation and reduced survival (Fig. 1L, N).

To establish target-specific therapeutic models, we screened tumor cell lines for NKG2DL expression, identifying MC38 colorectal carcinoma and Hepa1-6 HCC cells as high expressors, while H22 HCC and B16F10 melanoma cells showed low expression (Fig. 1O, Fig. S2A). We subsequently engineered a lentiviral CAR construct comprising: the CD8 signal peptide, NKG2D extracellular domain, CD28 hinge region, and FcγRI transmembrane/activation domains, co-expressed with EGFP via an E2A linker (Fig. 1P). Transduction of murine iBMDM and J774A.1 macrophages achieved successful CAR-M generation, with flow cytometry confirming high EGFP expression (Fig. 1Q) and significantly elevated surface NKG2D levels in CAR-Ms versus GFP-M controls (Fig. 1R, Fig. S2B-C).

### CAR-M exhibits functional polarization and target-specific antitumor efficacy

**Fig. 2:**
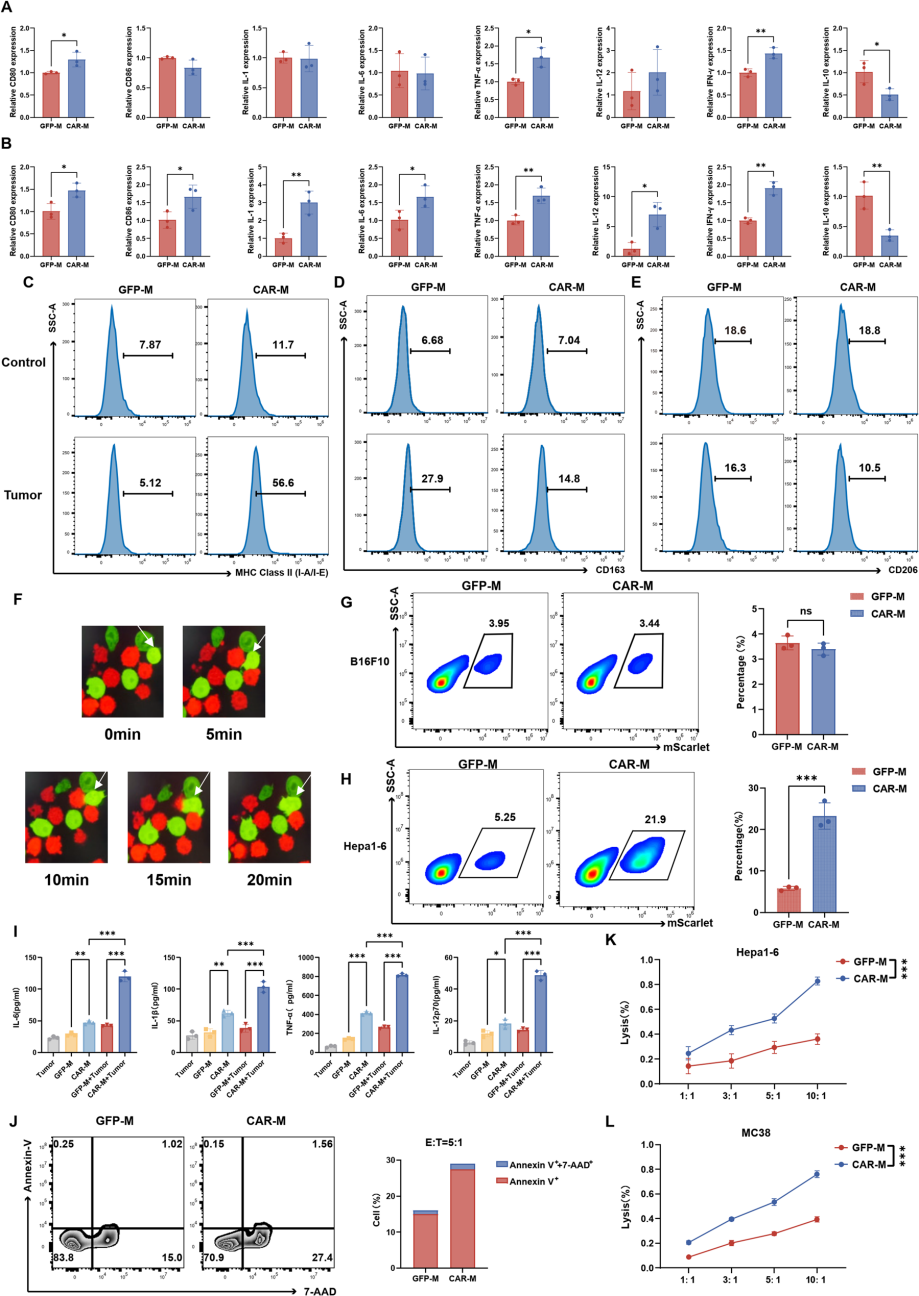
CAR-Ms exhibit enhanced phagocytic activity and tumoricidal polarization. **A**-**B** Quantitative PCR analysis of M1-associated markers (CD80, CD86, IL-1β, IL-6, TNF-α, IL-12, IFN-γ) and M2-associated marker IL-10 in (**A**) resting and (**B**) tumor-activated CAR-Ms versus GFP-M controls. **C**-**E** Flow cytometry analysis of (**C**) MHC-II and M2 markers (**D**) CD163 and (**E**) CD206 in CAR-Ms following 24-hour co-culture with Hepa1-6 cells (E: T ratio at 1:1). **F** Time-lapse imaging of a CAR-M (green) engulfing a Hepa1-6 tumor cell (red; arrow indicates phagocytic event). **G**-**H** Phagocytic efficiency of CAR-Ms against (**G**) NKG2DL-negative B16F10 and (**H**) NKG2DL-positive Hepa1-6 cells at 1:1 E: T ratio. **I** ELISA quantification of pro-inflammatory cytokines (IL-6, IL-1β, TNF-α, IL-12p70) in supernatants after 24-hour co-culture at 10:1 E: T ratio. **J** Apoptosis of Hepa1-6 cells co-cultured with CAR-Ms (E: T ratio at 10:1), assessed by Annexin V staining. **K**-**L** Luciferase-based cytotoxicity assay measuring the killing of (**K**) Hepa1-6 and (**L**) MC38 cells by CAR-Ms at a 10:1 E: T ratio over 24 h. All experiments were repeated at least three times independently. Data are presented as mean ± SD. *p < 0.05, **p < 0.01, ***p < 0.001

We first characterized the phenotypic plasticity of CAR-M under resting and tumor-engaged conditions. At baseline, CAR-Ms demonstrated upregulated co-stimulatory molecules (CD80) and pro-inflammatory cytokines (TNF-α, IFN-γ), coupled with reduced IL-10 expression compared to GFP-M controls (Fig. 2A). Tumor co-culture induced further enhancement of M1-associated markers including CD80/CD86, MHC-II and IL-1β/IL-6/TNF-α/IL-12/IFN-γ, while suppressing M2 markers CD163/CD206 (Fig. 2B-E), indicating tumor-triggered M1 polarization.

Functional assessment demonstrated that CAR-Ms engaged with tumor cells and exhibited target-selective phagocytic activity, as visualized by time-lapse imaging (Fig. 2F). Quantitative analysis showed that CAR-Ms demonstrated significantly higher phagocytic activity against NKG2DL-positive Hepa1-6 cells (21.9% phagocytosis) compared to NKG2DL-negative B16F10 cells (3.44%), while GFP-M controls showed minimal activity against both cell types (Fig. 2G-H). This target-selective phagocytic activity was associated with increased secretion of pro-inflammatory cytokines (IL-6, IL-1β, TNF-α, IL-12) following co-culture with tumor cells (Fig. 2I). The target-selective phagocytic effects were consistently observed in J774A.1-derived CAR-Ms as well (Fig. S3A).

Beyond direct phagocytosis, CAR-Ms induced tumor cell death in co-culture systems (Fig. 2J). Dose- and time-dependent cytotoxicity assays demonstrated significant elimination of NKG2DL-positive Hepa1-6 cells and MC38 cells (E: T ratio-dependent killing at 24–48 h; Fig. 2K, L, Fig. S3B, C). These findings collectively establish CAR-Ms as multifunctional effector cells capable of target-specific tumor clearance through coordinated phagocytic and cytotoxic mechanisms.

### CAR-M activates innate immunity via dual signaling pathways and synergizes adaptive antitumor response

**Fig. 3:**
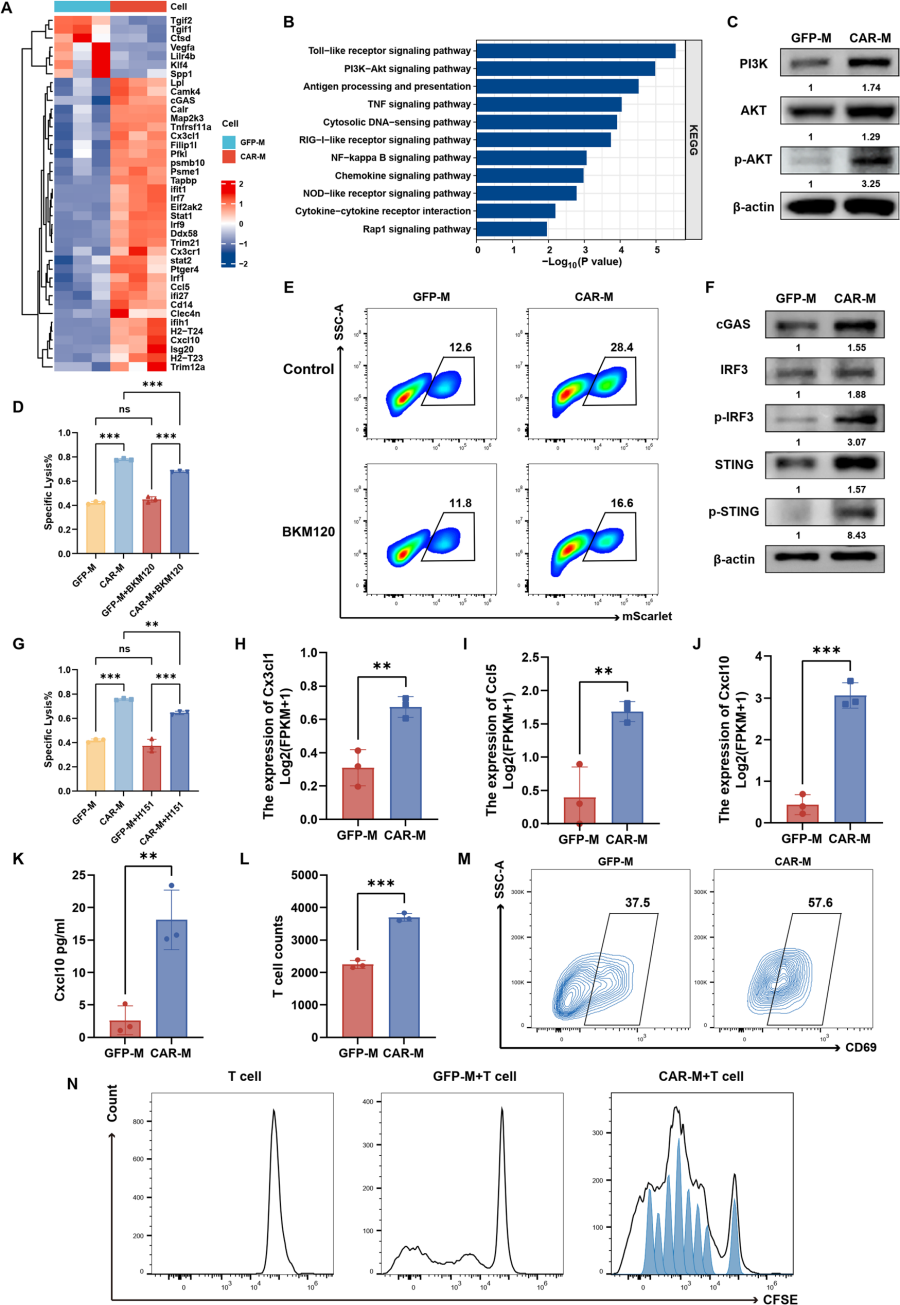
CAR-Ms activate PI3K-AKT and cGAS-STING pathways to enhance tumor clearance and promote T cell recruitment and activation. **A** Heatmap of differentially expressed genes in CAR-Ms versus GFP-M controls after tumor co-culture. **B** KEGG pathway enrichment analysis of genes upregulated in CAR-Ms. **C** Representative western blots of PI3K-AKT pathway proteins in CAR-Ms following tumor challenge. Values below lanes indicate relative protein expression (mean ± SD, *n* = 3), normalized to β-actin, with the GFP-M group set to 1. **D** Luciferase-based cytotoxicity of CAR-Ms against Hepa1-6 cells (E: T = 10:1) with or without PI3K inhibitor BKM120 (*n* = 3). **E** Phagocytic efficiency of CAR-Ms against Hepa1-6 cells (E: T = 1:1) following PI3K inhibition (*n* = 3). **F** Representative western blots of cGAS-STING pathway proteins in CAR-Ms. Values below lanes indicate relative protein expression (mean ± SD, *n* = 3), normalized to β-actin, with the GFP-M group set to 1. **G** Cytotoxicity of CAR-Ms against Hepa1-6 cells (E: T = 10:1) with or without the STING inhibitor H-151 (*n* = 3). **H**-**J** qRT-PCR analysis of T cell-attracting chemokines (Cx3cl1, Ccl5, Cxcl10) in CAR-Ms (*n* = 3). **K** ELISA quantification of Cxcl10 in co-culture supernatants (*n* = 3). **L** Transwell migration assay quantifying T cell recruitment by CAR-Ms (*n* = 3). **M**-**N** CAR-Ms enhance T cell activation and proliferation in macrophage-tumor-T cell tri-cultures, as assessed by (**M**) CD69 expression and (**N**) CFSE dilution (macrophage: tumor: T cell ratio = 2:1:2; *n* = 3 independent experiments). Data are presented as mean ± SD. *p < 0.05, **p < 0.01, ***p < 0.001

To elucidate the mechanisms underlying CAR-M-mediated tumor phagocytosis and killing, we analyzed transcriptional profiles of CAR-M and GFP-M after tumor co-culture. Differential gene expression analysis revealed that CAR-M exhibited marked upregulation of inflammatory response-related genes (e.g., Irf1, Irf7, Ddx58), antigen processing/presentation genes (H2-T24, H2-T23, Tapbp, Psme1), and chemokines (Cxcl10, Ccl5, Cx3cl1), alongside downregulation of M2 polarization markers (Klf4, Vegfa, SPP1) and tumor-promoting genes (Tgif1/2) compared to GFP-M (Fig. 3A). KEGG pathway enrichment analysis demonstrated that differentially expressed genes in CAR-M were predominantly enriched in inflammation-associated pathways, including Toll-like receptor, TNF, RIG-I-like receptor, and NF-κB, indicating robust tumor-triggered inflammatory activation (Fig. 3B, Fig. S4). Moreover, CAR-Ms are enriched with PI3K-AKT signaling pathways, antigen processing and presentation, and chemokine signaling pathways, suggesting that multiple anti-tumor mechanisms of CAR-M contribute to its phagocytic and cytotoxic effects on tumors (Fig. 3B, Fig. S4).

Mechanistically, transcriptional data indicated that the PI3K-AKT signaling pathway was significantly upregulated in CAR-M, which is reported to phosphorylate IKK to inactivate the inhibitory protein IκB, thereby releasing its suppression on NF-κB and ultimately activate NF-κB signaling pathway [17]. Indeed, western blot confirmed enhanced expression of PI3K, AKT, and phosphorylated AKT (p-AKT) following tumor co-culture (Fig. 3C). Pharmacological inhibition of PI3K with BKM120 substantially impaired CAR-M phagocytic and cytotoxic capacities (Fig. 3D, E, Fig. S5A, C, E). Furthermore, building on our prior finding that tumor-derived DNA escaping from phagolysosomes activates the cGAS-STING pathway in HER2-targeted CAR-M [18], we validated that NKG2DLs-targeted CAR-M similarly exhibited upregulated cGAS, STING, phosphorylated STING (p-STING), IRF3, and phosphorylated IRF3 (p-IRF3) post-tumor phagocytosis (Fig. 3F). STING inhibitor H-151 treatment significantly attenuated CAR-M-mediated tumor killing (Fig. 3G, Fig. S5B, D, F), confirming the synergistic roles of PI3K-AKT and cGAS-STING pathways in CAR-M innate immune functions.

Notably, CAR-M post-tumor stimulation exhibited elevated secretion of T/NK cell-recruiting chemokines (Cx3cl1, Ccl5, Cxcl10) (Fig. 3H–J), with Cxcl10 secretion further validated by ELISA (Fig. 3K). Transwell assays confirmed that activated CAR-M effectively recruited T cells (Fig. 3L). Enhanced MHC class I (H2-T24, H2-T23) and antigen processing (Psme1, Tapbp) gene expression in CAR-M suggested improved antigen presentation. In macrophage-tumor-T cell tri-cultures, CAR-M significantly promoted T cell activation and proliferation compared to GFP-M controls (Fig. 3M, N). Collectively, these findings demonstrate that CAR-M orchestrates dual antitumor mechanisms: direct tumor elimination via innate immune pathways (PI3K-AKT and cGAS-STING) and indirect adaptive immune activation through chemokine-driven T cell recruitment and antigen presentation, suggesting potent combinatorial therapeutic efficacy.

### Systemic CAR-M administration suppresses subcutaneous tumor growth and reprograms myeloid cell phenotypes

**Fig. 4:**
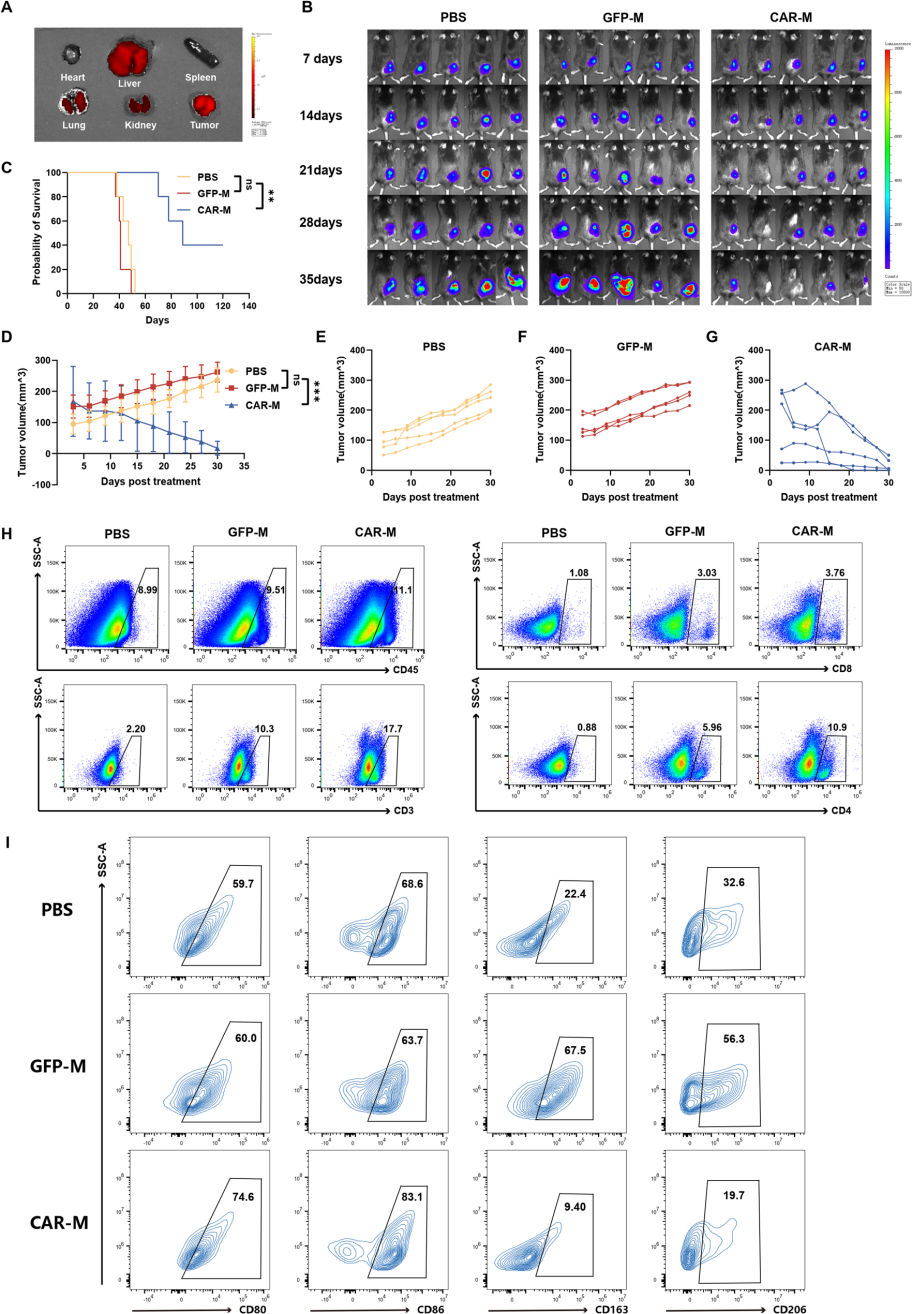
CAR-Ms suppress subcutaneous HCC growth and reprogram the immunosuppressive TME. **A** C57BL/6 mice bearing subcutaneous Hepa1-6 tumors (4 × 10⁶ cells) received a single intravenous injection of 1 × 10⁷ GFP-labeled CAR-Ms on day 14 post-implantation. In vivo distribution of CAR-Ms was assessed by ex vivo fluorescence imaging of harvested organs 35 days after CAR-M administration. **B** Subcutaneous Hepa1-6 tumors were established in C57BL/6 mice, followed by intravenous administration of PBS, GFP-M (2 × 10⁷ cells), or CAR-M (2 × 10⁷ cells) at day 7 post-implantation. Tumor growth was monitored via BLI at the indicated timepoints (*n* = 5 mice per group). **C** Kaplan-Meier survival for the subcutaneous Hepa1-6 cohorts shown in (**B**). **D** Tumor volume measurements across treatment groups (PBS, GFP-M, CAR-M) monitored at 3-day intervals. **E**-**G** Growth curves for individual mice in the (**E**) PBS-, (**F**) GFP-M-, and (**G**) CAR-M-treated groups. **H**-**I** Flow cytometry analysis of (**H**) tumor-infiltrating immune cells (CD45⁺, CD3⁺, CD4⁺, CD8⁺) and (**I**) myeloid cell polarization (M1: CD80⁺/CD86⁺; M2: CD163⁺/CD206⁺) in excised tumors at 5 days post-treatment initiation (*n* = 3 mice per group). Data are presented as mean ± SD. *p < 0.05, **p < 0.01, ***p < 0.001

To assess the therapeutic potential of CAR-M in vivo, we established subcutaneous Hepa1-6 HCC models in C57BL/6 mice (Fig. S6A). Following intravenous administration, GFP-labeled CAR-Ms were tracked at 35 days post-infusion and exhibited multiple tissue distribution. Notably, CAR-Ms were predominantly detected in the lungs, liver, and tumor tissues, with minimal presence in the kidneys and no significant accumulation in the heart or spleen (Fig. 4A).

Longitudinal bioluminescence imaging (BLI) revealed a marked suppression of tumor growth in CAR-M-treated mice compared to those receiving PBS or GFP-M controls (Fig. 4B, D–G). This antitumor effect was associated with a significant survival advantage, as CAR-M-treated mice exhibited prolonged overall survival relative to control groups (Fig. 4C).

We next profiled the tumor immune microenvironment by flow cytometry. CAR-M treatment led to a significant increase in the infiltration of CD45⁺ leukocytes, including CD3⁺ T cells (both CD4⁺ and CD8⁺ subsets) and B cells, compared to controls (Fig. 4H, Fig. S6B). Furthermore, CAR-M administration induced a phenotypic shift in tumor-associated myeloid cells. CD11b⁺ cells from CAR-M-treated tumors showed upregulated expression of M1-associated markers (CD80, CD86) and downregulated expression of M2-associated markers (CD163, CD206), indicating a repolarization toward an immunostimulatory phenotype (Fig. 4I).

Throughout the study, body weight remained stable across all groups, with no significant differences observed between CAR-M-treated and control mice (Fig. S6C). Histopathological evaluation of major organs (heart, liver, spleen, lung, kidney, brain) via H&E staining revealed no notable morphological abnormalities in CAR-M-treated mice compared to GFP-M controls, supporting the favorable safety profile of the treatment (Fig. S6D).

Together, these data demonstrate that systemically delivered CAR-M not only directly targets and suppresses tumor growth but also remodels the immunosuppressive tumor microenvironment by enhancing immune cell infiltration and promoting pro-inflammatory myeloid polarization, thereby orchestrating a potent antitumor immune response.

### CAR-M induces durable tumor regression in orthotopic liver cancer models

**Fig. 5:**
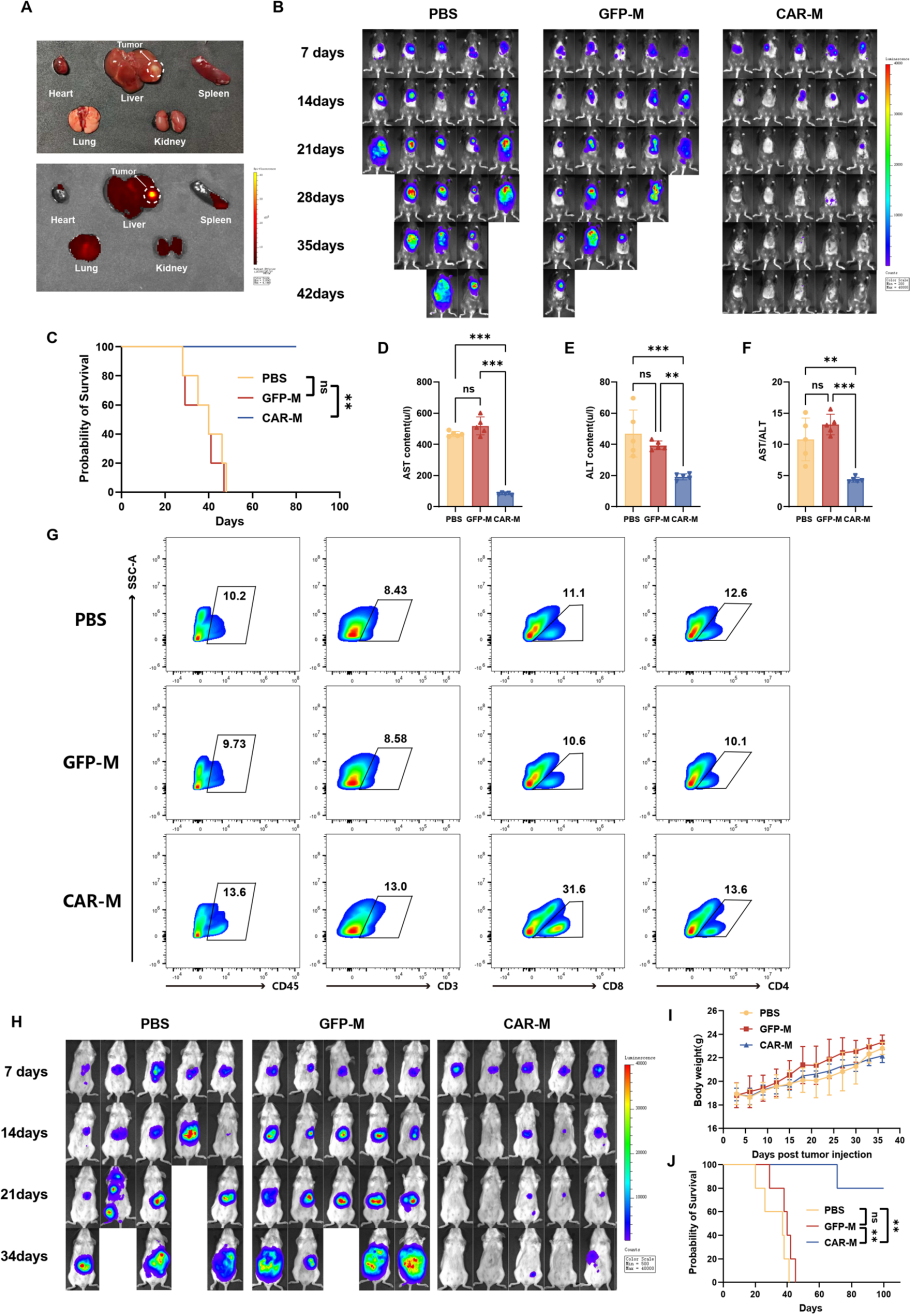
CAR-Ms demonstrate therapeutic efficacy in orthotopic HCC models. **A** In vivo distribution of CAR-Ms was evaluated by ex vivo fluorescence imaging of harvested organs 20 days after intravenous administration of 1 × 10⁷ GFP-labeled cells into mice bearing orthotopic Hepa1-6 tumors, with the top and bottom panels showing the resected organs under visible light and fluorescence, respectively. **B** Orthotopic Hepa1-6 HCC tumors were established in C57BL/6 mice. At day 7 post-implantation, mice received intravenous injections of PBS, GFP-M (2 × 10⁷ cells), or CAR-M (2 × 10⁷ cells). Tumor burden was monitored by BLI every 7 days (*n* = 5 mice per group). **C** Kaplan-Meier survival curves of mice bearing orthotopic Hepa1-6 tumors. **D**-**F** Serum levels of AST (**D**), ALT (**E**), and AST/ALT ratio (**F**) at 4 weeks post-treatment. **G** Tumor-infiltrating immune cells were analyzed by flow cytometry for expression of CD45, CD3, CD4, and CD8. **H** BALB/c mice received hepatic subcapsular injection of H22-Rae-1β cells (1 × 10⁶) to establish an orthotopic tumor model. On day 7, mice were treated intravenously with PBS, GFP-J774A.1 (5 × 10⁶ cells), or CAR-J774A.1 (5 × 10⁶ cells). Tumor burden was assessed by BLI (*n* = 5 mice per group). **I**-**J** Body weight changes (**I**) and survival analysis (**J**) for the H22-Rae-1β orthotopic model cohorts. Data are presented as mean ± SD. *p < 0.05, **p < 0.01, ***p < 0.001

To better recapitulate the clinical progression of HCC, we established an orthotopic model by implanting Hepa1-6 tumor tissues directly into the livers of C57BL/6 mice (Fig. S7A). Following 20 days after intravenous administration, GFP-labeled CAR-Ms were tracked in vivo. Similar to the subcutaneous model, CAR-Ms were predominantly localized in the lungs and liver, with moderate accumulation also observed in the kidneys. Notably, CAR-Ms showed significantly higher enrichment in orthotopic liver tumors than in any other organ, indicating a specific tropism for hepatic tumor tissues (Fig. 5A).

Tumor-bearing mice received a single intravenous injection of PBS, GFP-M, or CAR-M seven days post-implantation. CAR-M treatment resulted in profound suppression of tumor progression. While mice in the PBS and GFP-M control groups exhibited progressive tumor enlargement and succumbed to the disease by week 6, CAR-M-treated mice displayed rapid tumor regression beginning at week 2, culminating in complete tumor eradication by week 5. All CAR-M-treated mice survived until the experimental endpoint without evidence of tumor recurrence (Fig. 5B, C).

No significant differences in body weight were observed among the groups throughout the study period (Fig. S7B). Serum biochemical analysis indicated that CAR-M-treated mice exhibited the lowest levels of liver injury markers (AST and ALT) as well as AST/ALT ratios, suggesting better preservation of hepatic function compared to controls (Fig. 5D–F). Flow cytometric analysis of tumor-derived single-cell suspensions revealed significantly increased infiltration of CD45⁺ immune cells, CD3⁺ T cells, and both CD4⁺ and CD8⁺ T cell subsets in the CAR-M group (Fig. 5G). Immunohistochemistry further confirmed enhanced signals for caspase 3 (apoptosis marker), CD3, CD8, and EGFP (for CAR-M tracking) in CAR-M-treated tumors, underscuring robust CAR-M infiltration, T cell recruitment, and apoptotic activity (Fig. S7C).

To validate these findings across different experimental contexts, we developed an alternative orthotopic HCC model in BALB/c mice using Rae-1–overexpressing H22 cells (Fig. S8A–C). Consistent with the results in C57BL/6 mice, administration of J774A.1-derived CAR-Ms significantly suppressed tumor growth and extended survival compared to PBS and GFP-M controls (Fig. 5H–J).

Collectively, these results demonstrate that CAR-M treatment drives potent and durable tumor regression in orthotopic liver cancer models through direct tumoricidal activity and coordinated immune activation, leading to significantly improved survival outcomes across multiple murine backgrounds.

### CAR-M suppresses metastatic HCC in peritoneal and pulmonary models via targeted elimination of disseminated lesions

**Fig. 6:**
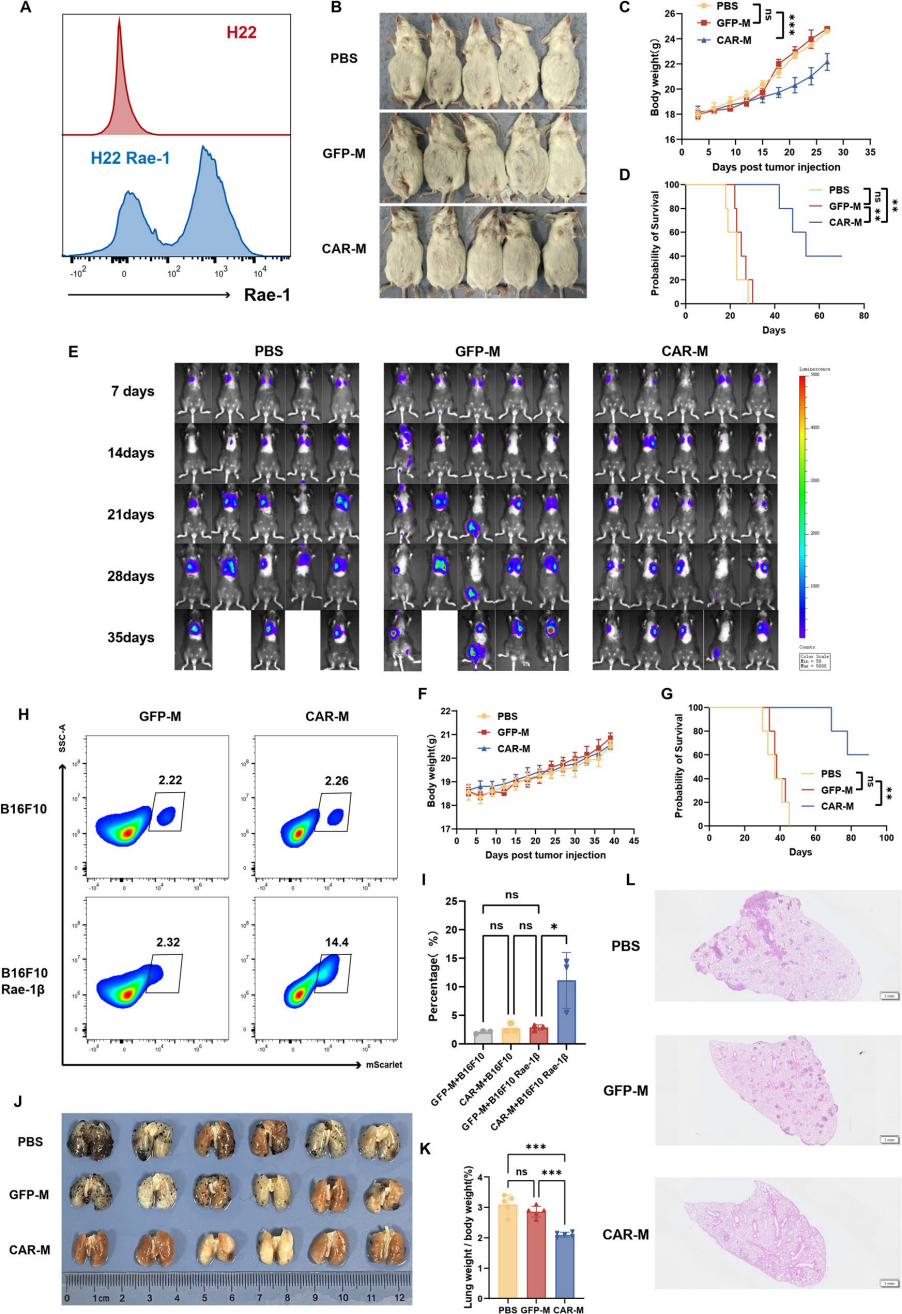
CAR-Ms target metastatic HCC in peritoneal and pulmonary models. **A** Flow cytometry analysis of Rae-1β expression in H22 and H22-Rae-1β cells. **B** BALB/c mice were intraperitoneally injected with H22-Rae-1β cells (1 × 10⁵). On day 7, mice received intraperitoneal administration of PBS, GFP-J774A.1 (1 × 10⁶ cells), or CAR-J774A.1 (1 × 10⁶ cells). Ascites progression was monitored over 2 weeks (*n* = 5 mice per group). **C**-**D** Longitudinal body weight changes (**C**) and Kaplan-Meier survival analysis (**D**) of mice in the peritoneal dissemination model. **E** C57BL/6 mice were intravenously injected with Hepa1-6 cells (4 × 10⁶). At day 7, mice were treated intravenously with PBS, GFP-M (2 × 10⁷ cells), or CAR-M (2 × 10⁷ cells). Tumor burden was monitored by BLI at 7-day intervals (*n* = 5 mice per group). **F**-**G** Longitudinal body weight tracking (**F**) and Kaplan-Meier survival analysis (**G**) in pulmonary metastasis cohorts. **H**-**I** Phagocytic activity of CAR-Ms against B16F10 (NKG2DL-negative) and B16F10-Rae-1β (NKG2DL-positive) cells at 1:1 E: T ratio, as analyzed by flow cytometry (**H**) and corresponding quantification (**I**). Data are representative of three independent experiments. **J**-**L** C57BL/6 mice received intravenous injection of B16F10-Rae-1β cells (2 × 10⁵) followed by CAR-M treatment (*n* = 6 mice per group). **J** Macroscopic evaluation of lung metastases. **K** Quantification of lung weight to body weight ratio. **L** Representative H&E-stained lung sections for visualization of metastatic foci. Data are presented as mean ± SD. *p < 0.05, **p < 0.01, ***p < 0.001

Metastasis represents a leading cause of mortality in HCC. To evaluate the potential of CAR-M in controlling metastatic disease, we established both peritoneal dissemination and pulmonary metastasis models. For the peritoneal model, we employed two H22 cell variants: NKG2D^low^ H22 cells and H22 cells overexpressing a truncated form of Rae-1β (H22-Rae-1β) (Fig. 6A). In mice bearing H22-Rae-1β tumors, intraperitoneal (i.p.) administration of CAR-M seven days post-inoculation (Fig. S9A) led to significant suppression of tumor progression, as indicated by reduced ascites volume, attenuated body weight gain associated with ascites (Fig. 6B, C), and prolonged survival compared to PBS and GFP-M control groups (Fig. 6D). A similar therapeutic benefit was observed in the NKG2D^low^ H22 peritoneal model, where CAR-M treatment delayed hemorrhagic ascites formation, ameliorated associated body weight increases (Fig. S9B–E), and significantly extended survival (Fig. S9F), confirming its potent locoregional antitumor activity.

We next assessed CAR-M efficacy in a hematogenous spread model by intravenously injecting Hepa1-6 cells to establish pulmonary metastases. Intravenous administration of CAR-M seven days post-tumor inoculation (Fig. S10A) markedly inhibited the progression of lung metastases, resulting in fewer and smaller metastatic nodules and significantly prolonged survival compared to controls (Fig. 6E–G).

To further validate the specificity of CAR-M targeting, we engineered B16F10 melanoma cells to overexpress Rae-1β (B16F10-Rae-1β; Fig. S10B). Consistent with the expected mechanism, CAR-M exhibited significantly enhanced phagocytic capacity against B16F10-Rae-1β cells compared to native B16F10 cells, which express low levels of NKG2D ligands (Fig. 6H, I). In a B16F10-Rae-1β pulmonary metastasis model (Fig. S10C), CAR-M treatment substantially reduced lung metastatic burden (Fig. 6J) and the lung weight/body weight ratio (Fig. 6K). Histopathological examination further confirmed the near-complete absence of metastatic lesions in the lungs of CAR-M-treated mice (Fig. 6L).

Collectively, these findings demonstrate that CAR-M effectively targets and eliminates disseminated tumor cells in both peritoneal and pulmonary metastatic niches, underscoring its potential as a promising therapeutic strategy against locoregional and distant metastasis in advanced HCC.

### CAR-M therapy establishes tumor-specific immunological memory and prevents tumor relapse

**Fig. 7:**
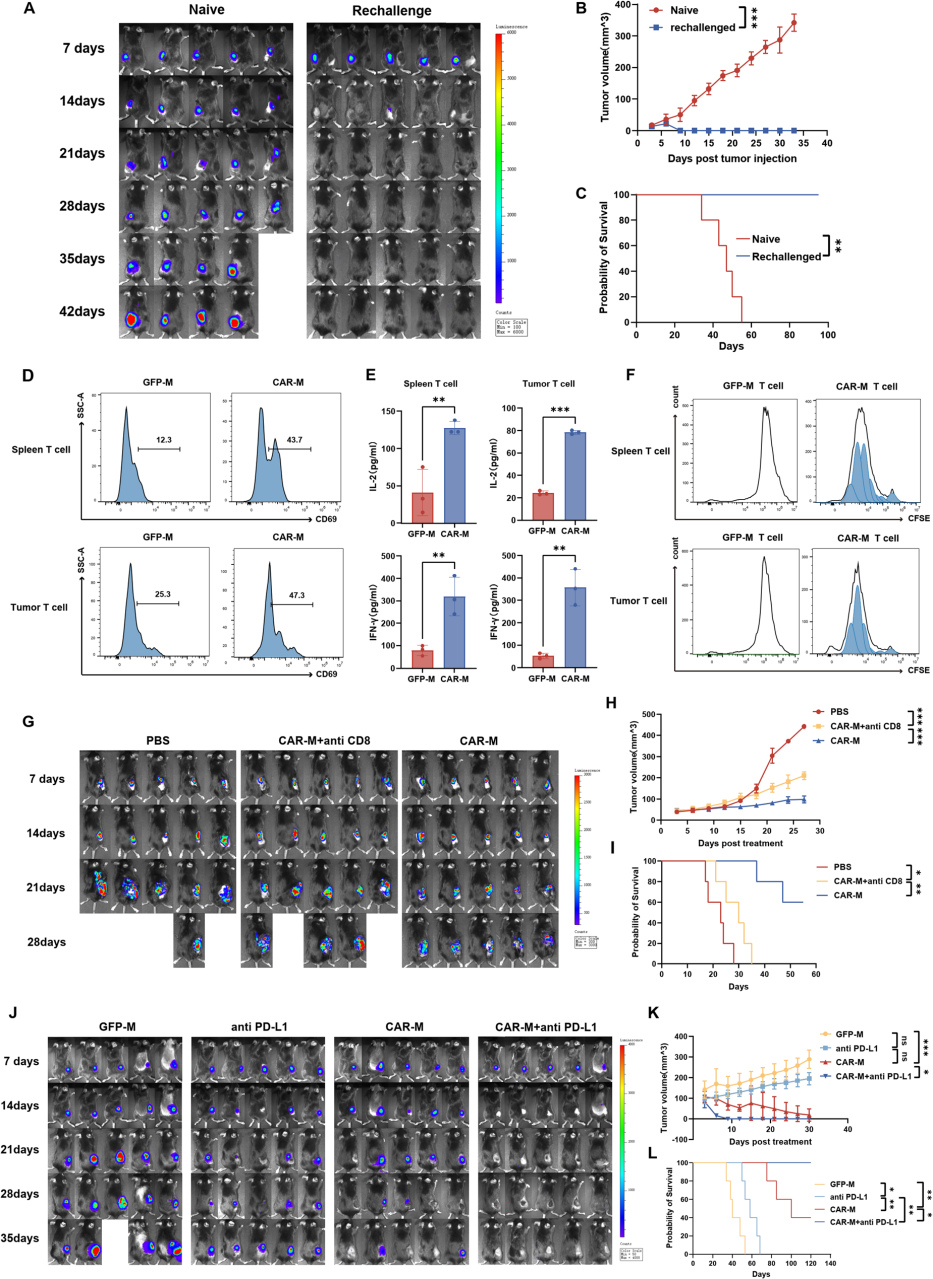
CAR-M combined with PD-L1 blockade enhances antitumor efficacy and promotes immune memory. **A** C57BL/6 mice with established subcutaneous Hepa1-6 tumors received intravenous CAR-M treatment (2 × 10⁷ cells) on day 7. Following complete tumor regression, mice were rechallenged with Hepa1-6 cells in the contralateral flank. Tumor growth was monitored via BLI at the indicated timepoints (**B**) Tumor growth kinetics and (**C**) Kaplan-Meier survival analysis of naïve and CAR-M-cured mice after tumor rechallenge (*n* = 5 mice per group). **D**–**F** T cells isolated from spleens and tumors of CAR-M- or GFP-M-treated mice were co-cultured with Hepa1-6 cells. Tumor-specific T cell responses were assessed by (**D**) CD69 expression (flow cytometry), (**E**) IL-2 and IFN-γ secretion (ELISA), and (**F**) proliferation (CFSE dilution) (*n* = 3 mice per group). **G** C57BL/6 mice were subcutaneously implanted with B16-Rae-1β cells (3 × 10⁵) and treated intravenously at day 7 with PBS, CAR-M (3 × 10⁶ cells) plus anti-CD8 depleting antibody, or CAR-M alone. Tumor burden was assessed by BLI at 7-day intervals. **H** Tumor growth curves and **I** survival analysis following CD8⁺ T cell depletion in CAR-M-treated mice (*n* = 5 mice per group). **J** Mice with subcutaneous Hepa1-6 tumors received the indicated monotherapies or combination treatment starting on day 7. Tumor burden was assessed by BLI at 7-day intervals. **K** Tumor growth kinetics and (**L**) survival analysis across treatment groups (*n* = 5 mice per group). Data are presented as mean ± SD. *p < 0.05, **p < 0.01, ***p < 0.001

To investigate whether CAR-M treatment induces long-term antitumor immunity, we performed tumor rechallenge experiments in mice that had achieved complete regression of subcutaneous Hepa1-6 tumors following CAR-M therapy. These tumor-free mice, along with naïve controls, were rechallenged with Hepa1-6 cells in the contralateral flank. While control mice developed rapidly progressing tumors, all CAR-M-cured mice effectively cleared the reinoculated tumor cells within one week (Fig. 7A, B). Remarkably, 100% of the rechallenged mice remained tumor-free for nearly 100 days, whereas all naïve control mice succumbed to tumor growth within two months (Fig. 7C).

To assess whether this protective effect was mediated by tumor-specific immune memory, we isolated T cells from the spleens and tumor tissues of CAR-M-treated mice. Upon co-culture with Hepa1-6 cells, these T cells exhibited significantly higher expression of the early activation marker CD69 (Fig. 7D), secreted increased levels of IL-2 and IFN-γ (Fig. 7E), and showed enhanced proliferative capacity (Fig. 7F) compared to those from control mice, indicating the establishment of robust tumor-specific T cell immunity.

To functionally validate the contribution of T cells to this sustained antitumor response, we depleted CD8⁺ T cells in CAR-M-treated tumor-bearing mice using a neutralizing antibody. T cell depletion resulted in significantly increased tumor burden (Fig. 7G, H) and shortened survival (Fig. 7I), confirming that CD8⁺ T cells are essential for maintaining the long-term antitumor efficacy of CAR-M therapy.

Together, these results demonstrate that CAR-M not only directly eliminates tumor cells but also orchestrates a durable adaptive immune response, enabling the establishment of tumor-specific immunological memory that protects against tumor recurrence.

### CAR-M combined with PD-L1 blockade results in enhanced antitumor efficacy

Immune checkpoint blockade, particularly targeting the PD-1/PD-L1 axis, has shown clinical utility in hepatocellular carcinoma (HCC), but its efficacy is often constrained by insufficient T cell infiltration and an immunosuppressive tumor microenvironment. Given the capacity of CAR-M to remodel the tumor immune landscape and promote T cell recruitment, we hypothesized that combining CAR-M with PD-L1 inhibition could enhance antitumor efficacy. We first confirmed substantial PD-L1 expression on several mouse tumor cell lines, including GL261, Hepa1-6, and MC38 (Fig. S11A). Furthermore, co-culture of macrophages with tumor cells led to upregulated PD-1 and PD-L1 expression on macrophages (Fig. S11B). Functionally, the addition of an anti-PD-L1 antibody enhanced the phagocytic capacity of CAR-M against tumor cells in vitro (Fig. S11C, D), suggesting that PD-L1 blockade may potentiate immune activation by both alleviating T cell exhaustion and directly enhancing macrophage function.

To evaluate the therapeutic potential of combining CAR-M with PD-L1 blockade in vivo, we established subcutaneous Hepa1-6 tumors in C57BL/6 mice (Fig. S12A). CAR-M and anti-PD-L1 antibody were administered individually or in combination beginning on day 7 post-tumor inoculation. Both monotherapies significantly suppressed tumor growth and extended survival; however, the combination treatment resulted in a markedly enhanced antitumor effect, leading to accelerated and complete tumor eradication within one week (Fig. 7J, K; Fig. S12B-E). Consequently, mice receiving the combined regimen achieved a significantly greater survival advantage over those receiving either monotherapy (Fig. 7L). No notable differences in body weight were observed among the groups (Fig. S12F), indicating that the combination was well tolerated.

Immunohistochemistry and immunofluorescence analysis of tumor tissues revealed that CAR-M monotherapy increased the levels of activated caspase-3, as well as the infiltration of CD3⁺ T cells, CD8⁺ T cells, and GFP-labeled CAR-Ms relative to controls. These effects were most pronounced in the combination treatment group (Fig. S12G). Flow cytometry analysis of tumor-infiltrating lymphocytes further showed a stepwise increase in activated T cells (CD3⁺CD69⁺), cytokine-producing cytotoxic T cells (CD3⁺TNF-α⁺/IFN-γ⁺), and CD3⁺PD-1⁺ T cells across treatment groups (anti-PD-L1 < CAR-M < combination), a trend that correlated well with the observed therapeutic efficacy and survival outcomes (Fig. S12H).

Collectively, these data indicate that the combination of CAR-M with PD-L1 blockade enhances antitumor immunity by concurrently boosting innate phagocytic activity and potentiating adaptive T cell responses, resulting in superior therapeutic outcomes compared to monotherapy.

## Discussion

While CAR-T therapy holds promise for HCC, its clinical translation is hindered by tumor infiltration barriers, immunosuppressive TME, antigenic heterogeneity, and T cell exhaustion [19–21]. In contrast, macrophages exhibit unique advantages, including exceptional tumor tissue penetration capacity and high adaptability to hypoxic microenvironments [14, 15]. Seminal work by Morrissey et al. in 2018 established CAR-Ms, demonstrating that Megf10/FcγR cytoplasmic domains trigger antigen-dependent phagocytosis [22], with subsequent validation by Klichinsky et al. confirming efficacy against HER2-positive solid tumors [12]. Recent advances have propelled CAR-M development across multiple technical dimensions, including but not limited to: (1) diversification of cellular sources (e.g. primary monocytes, macrophage cell line, and iPSC-derived macrophages) [12–25]; (2) structural innovations aimed at augmenting antitumor efficacy [18, 26–28]; (3) rational combination with immune checkpoint inhibitors [18, 29]; and (4) in situ engineering for localized activation [30–32]. Notably, the ongoing Phase I trial NCT04660929 reported preliminary safety and disease stabilization in 28.6% (2/7) of HER2 3^+^ patients treated with HER2-CAR-Ms (CT-0508), underscoring clinical feasibility [20]. Despite these advancements, CAR-M applications in HCC remain underexplored, necessitating further mechanistic adaptations tailored to liver malignancies.

Structurally, CAR-M retains the modular architecture of CAR-T constructs, comprising an antigen-binding domain, transmembrane region, and intracellular signaling domains [33]. While most studies employ scFvs for tumor antigen recognition, endogenous receptor-ligand pairs offer advantages including enhanced structural stability, reduced immunogenicity, and evolutionary optimization. Our work focuses on NKG2D—a C-type lectin-like receptor (CTLR) expressed on NK cells, CD8^+^ T cells, and γδ T cells—that recognizes stress-induced ligands (NKG2DLs) upregulated during malignant transformation, DNA damage, or viral infection. In humans, NKG2DLs encompass MICA, MICB, and ULBP1-6, while murine counterparts include Rae-1 and H60 families, and Mult1. Bioinformatics analysis of HCC clinical specimens and tissue microarrays revealed significant NKG2DL overexpression in tumors compared to adjacent non-tumor tissues, correlating with poorer patient survival—a finding supporting NKG2DLs as compelling therapeutic targets. Notably, NKG2D-based CAR-T therapies have demonstrated efficacy across multiple cancers [34–36], with theoretical advantages for mitigating antigen escape due to the polyvalent nature of NKG2DL induction under tumor stress.

Our in vitro studies elucidated the mechanistic basis of NKG2DL-targeted CAR-Ms as multifunctional antitumor effector cells. These engineered CAR-Ms selectively engulfed NKG2DL-positive tumor cells through FcγRI-mediated phagocytosis while simultaneously secreting pro-inflammatory cytokines (e.g., TNF-α, IL-12), a dual functional output driven by coordinated activation of the PI3K-AKT and cGAS-STING signaling pathways. Transcriptional profiling further revealed CAR-M-driven upregulation of T cell-recruiting chemokines (Cxcl10, Ccl5), MHC class I molecules (H2-T23/24), and antigen-processing machinery components (Tapbp, Psme1). This molecular reprogramming enabled two key outcomes: (1) robust recruitment of effector T cells to tumor niches via chemokine gradients, and (2) efficient cross-presentation of tumor antigens via MHC class I molecules to prime polyclonal CD8^+^ T cell responses. Collectively, these mechanisms established a functional bridge between innate tumor clearance and adaptive immune activation. Critically, the combination of polyclonal CD8^+^ T cell responses and polyvalent antigen recognition by NKG2D directly targets the fundamental challenges of tumor heterogeneity and recurrence in HCC therapy, offering a solution to overcome therapeutic resistance.

The choice of immunocompetent murine models for in vivo evaluation provided critical insights into CAR-M-mediated immune remodeling. Unlike immunodeficient systems, these models preserved intact lymphoid-myeloid interactions, revealing CAR-M’s ability to recruit CD45^+^ leukocytes, CD3^+^ T cells, and B cells into tumors while converting myeloid cells from an immunosuppressive M2 phenotype (CD163^+^/CD206^+^) to an immunostimulatory M1 state (CD80^+^/CD86^+^). This reprogramming effectively transformed “cold” tumors into “hot” niches, characterized by heightened T cell infiltration and enhanced antigen-presenting capacity—a prerequisite for overcoming HCC’s immunosuppressive barriers. Importantly, the observed TME remodeling was not merely a bystander effect but a direct consequence of CAR-M’s ability to simultaneously eliminate tumor cells and secrete chemotactic signals like Cxcl10, which recruits CXCR3^+^ T cells to establish an inflamed microenvironment.

A key finding from our in vivo studies was the superior efficacy of CAR-Ms in orthotopic liver models compared to subcutaneous models. Our tracking data indicated that intravenously infused CAR-Ms exhibited a specific tropism for liver tissues and showed significantly higher enrichment in orthotopic liver tumors than in any other organ, including subcutaneous tumors. This preferential homing to the liver likely underlies the more potent tumor regression and complete cures observed in the orthotopic setting, highlighting the importance of the tissue microenvironment for CAR-M efficacy.

The therapeutic efficacy of CAR-Ms in vivo emerges from a self-reinforcing cycle that integrates innate and adaptive immunity. Direct tumor phagocytosis by CAR-Ms initiates antigen release, which—combined with their upregulated MHC-I and antigen-processing machinery—primes tumor-specific T cell responses. Our data demonstrate that T cells from CAR-M-cured mice mounted robust tumor-specific responses upon re-exposure to tumor cells, characterized by rapid activation (CD69), proliferation, and effector cytokine (IL-2, IFN-γ) production (Fig. 7D-F). Activated T cells, in turn, secrete IFN-γ to sustain CAR-M M1 polarization, further enhancing chemokine production (e.g., Cxcl10) and antigen presentation. The critical role of this adaptive immune response was unequivocally demonstrated by CD8^+^ T cell depletion experiments, which significantly abrogated the antitumor efficacy of CAR-M therapy (Fig. 7G-I), confirming that durable remission depends on the engagement of the adaptive immune system. This feedforward loop not only amplifies immediate tumor clearance but also establishes durable immunologic memory, as evidenced by complete tumor rejection in rechallenged mice. Such synergy between CAR-M’s innate phagocytic activity and T cell-mediated adaptive immunity addresses a key limitation of conventional therapies, which often fail to concurrently target tumor cells and immunosuppressive circuits.

Our exploration of combination therapy revealed that the antitumor efficacy of CAR-Ms was significantly enhanced when combined with PD-L1 blockade. By recruiting T cells into tumors, CAR-Ms create a microenvironment permissive to PD-L1 inhibition. The combined treatment resulted in accelerated tumor clearance and superior survival outcomes compared to either monotherapy. We posit that this enhanced efficacy arises from a dual reinforcement mechanism: PD-L1 blockade likely disrupts PD-1/PD-L1-mediated inhibitory signals on both T cells and macrophages, thereby simultaneously reinvigorating exhausted T cells and potentially augmenting CAR-M phagocytic function, as suggested by our in vitro data. This reciprocal amplification of innate and adaptive arms of immunity represents a promising multimodal strategy for HCC.

It is also important to consider the translational implications of our target choice. While the restricted expression of NKG2DLs on tumors underpins their therapeutic appeal, their known induction on stressed or inflamed non-malignant tissues presents a theoretical risk of on-target, off-tumor toxicity. In our preclinical models, systemic CAR-M administration was well-tolerated, with no significant tissue damage detected by histopathology or serum biochemistry. However, these findings warrant cautious interpretation, and thorough safety profiling in more advanced models that better recapitulate human tissue stress and immune contexts remains an essential prerequisite for clinical translation.

Our comprehensive preclinical evaluation across diverse HCC models establishes that NKG2D-directed CAR-Ms achieve robust antitumor efficacy, characterized by profound tumor burden reduction, metastasis suppression, and survival prolongation. The superior performance of this strategy can be attributed to its multifunctional design: (1) Hepatic tropism—leveraging macrophages’ intrinsic liver-homing capacity for targeted tumor delivery, consistent with prior reports of CAR-Ms migration to hepatic niches [12, 18, 37]; (2) Polyvalent ligand recognition—NKG2D’s broad-spectrum targeting of stress-induced ligands combined with FcγRI-mediated phagocytic activation; (3) Coordinated immune recruitment—chemokine-driven T cell recruitment (e.g., Cxcl10) coupled with enhanced antigen cross-presentation; (4) Myeloid reprogramming—polarization of immunosuppressive CD11b^+^ cells toward immunostimulatory phenotypes; and (5) Durable immune memory—complete protection against tumor rechallenge through tumor-specific T cell immunity. These mechanistically interlinked functions enable NKG2D-CAR-Ms to simultaneously dismantle two pillars of HCC resistance: antigenic heterogeneity and immunosuppressive microenvironmental barriers. Importantly, the enhanced efficacy of combining CAR-M activity with PD-L1 blockade demonstrates a translatable strategy to counter adaptive immune evasion in advanced HCC, positioning this approach as a promising therapeutic platform worthy of further investigation.

## Conclusions

NKG2D-directed CAR-Ms eliminate HCC via coordinated innate phagocytosis and adaptive immune activation, driven by PI3K-AKT/cGAS-STING signaling. The engineered CAR-Ms not only directly clear tumor cells but also remodel the immunosuppressive TME through M1 polarization, T cell recruitment, and enhanced antigen presentation, leading to robust tumor regression and durable survival across multiple preclinical models. A key feature of this therapy is its ability to initiate a self-sustaining cycle of immunity: CAR-M phagocytosis primes tumor-specific T cell responses, which are essential for long-term efficacy as demonstrated by CD8^+^ T cell depletion, and these activated T cells in turn sustain CAR-M activity. Furthermore, combination with PD-L1 blockade results in enhanced antitumor efficacy by concurrently potentiating both innate phagocytic and adaptive cytotoxic arms of the immune response. This multifaceted strategy, which leverages macrophage tropism for liver tumors and polyvalent targeting of stress ligands, effectively addresses the dual challenges of antigenic heterogeneity and immunosuppression in HCC, presenting a promising and innovative immunotherapeutic approach worthy of further clinical investigation.

## Supplementary Information

Supplementary Material 1.

## Data Availability

The data that support the ﬁndings of this study are available from the corresponding author on request.

## Abbreviations

CAR-M: Chimeric antigen receptor macrophage
cGAS-STING: Cyclic GMP-AMP synthase-stimulator of interferon genes
FcγRI: Fc gamma receptor I
MICA/B: MHC class I chain-related protein A/B
NKG2D: Natural killer group 2D receptor
NKG2DL: NKG2D ligand
PI3K-AKT: Phosphatidylinositol 3-kinase-protein kinase B
Rae-1β: Retinoic acid early inducible-1β
scFv: Single-chain variable fragment
Tapbp: TAP-binding protein
ULBP: UL16-binding protein
