# Supplementary material for: Synergistic innate-adaptive immunity by NKG2D-specific CAR-macrophages drives durable remission in hepatocellular carcinoma
Source: Mol Cancer. 2025 Dec 13;25:9. doi: 10.1186/s12943-025-02538-w (PMC12784537; doi:10.1186/s12943-025-02538-w)
Supplement: Supplementary file 1 — Supplementary Material 1. [file 12943_2025_2538_MOESM1_ESM.docx]

Supplementary Materials for

**Synergistic Innate-Adaptive Immunity by NKG2D-Specific CAR-Macrophages Drives Durable Remission in Hepatocellular Carcinoma**

Zihao Zhao, Wenjing Zheng, Yang He, Han Zhang, Lingling Zhang, Yi Huo, Junwei Jiang, Haohan Lyu, Chen Liu, Feng Chang, Lequn Shan, Tao Wang, Wenjie Song

**This file includes:**

Supplementary Materials and Methods

Supplementary Figures 1 to 12

**Supplementary Materials and Methods**

**Cytokine Analysis**

Cytokine levels in cell culture supernatants were quantified using ELISA kits according to the manufacturer’s instructions. Mouse IL-1β (EK201B), TNF-α (EK282), IL-6 (EK206P), and IL-12p70 (EK212P) were measured with ELISA kits from Multi Sciences (Hangzhou, China). Mouse IL-2 (EK0398), IFN-γ (EK0375) were measured with ELISA kits from Boster (Wuhan, China). Briefly, 100 μL of supernatant or standard was added to pre-coated 96-well plates and incubated for 2 hours at room temperature. Detection antibodies and avidin-HRP were sequentially applied, followed by TMB substrate development. Absorbance was read at 450 nm using a Synergy H1 microplate reader (BioTek). Cytokine concentrations were calculated based on standard curves normalized to control groups.

**Western Blot Analysis**
Control and CAR-modified macrophages were co-cultured with Hepa1-6 tumor cells (E:T ratio 10:1) for 24 hours. Tumor-stimulated macrophages were isolated via FACS or immunomagnetic bead separation. Protein extraction used RIPA buffer supplemented with protease inhibitors, followed by BCA quantification. Equal amounts of protein (40 μg/cell lysates, 60 μg/tissue lysates) were resolved on 7.5% SDS-PAGE gels and transferred to PVDF membranes. Membranes were blocked with non-fat milk and incubated overnight with primary antibodies against PI3K(Proteintech, 20584-1-AP), AKT (Proteintech, 10176-2-AP), phospho-AKT (Cell Signaling Technology, 4060S), cGAS (Proteintech, 29958-1-AP), IRF3 (Proteintech, 66670-1-Ig), phospho-IRF3 (Proteintech, 29528-1-AP), STING (Proteintech, 19851-1-AP), phospho-STING (Cell Signaling Technology, 50907T), or beta-Actin (Cell Signaling Technology, 4970S). HRP-conjugated secondary antibodies were applied, and signals were detected using ECL reagent on a ChemiDoc imaging system. Band intensities were normalized to beta-Actin controls via Image Lab software.

**Transwell Migration** **Assay**

GFP-M or CAR-M (1×10⁴ cells/well) were seeded in 12-well plates and cultured for 24 hours to allow adherence. Concurrently, mouse splenic T cells were isolated using EasySep™ Mouse T Cell Isolation Kits (STEMCELL Technologies, 19851A) according to the manufacturer’s instructions. Purified T cells were subjected to serum starvation in RPMI-1640 medium without serum for 24 hours to synchronize their migratory state. Following starvation, T cells were resuspended at 1×10⁵ cells/mL in serum-free RPMI-1640.

For migration assays, 200 μL of T cell suspension was added to the upper chamber of Transwell inserts (5 μm pore size, Corning) pre-equilibrated with culture medium. Lower chambers contained 600 μL of conditioned medium from GFP-M or CAR-M cultures. Transwell plates were incubated at 37 °C for 24 hours. Migrated T cells in the lower chamber were collected, washed with PBS. Cell migration was quantified using BD FACS Canto™ II (BD Biosciences) with FlowJo v10.8.1 analysis. Experiments were performed in triplicate and repeated independently three times.

**Immunohistochemistry (IHC) and Immunofluorescence (IF)**
Formalin-fixed, paraffin-embedded tissue sections were deparaffinized in xylene, rehydrated through graded ethanol, and subjected to antigen retrieval via microwave heating (citrate/EDTA buffer, pH 6.0/9.0). Endogenous peroxidase activity was blocked with 3% H₂O₂ in methanol, followed by blocking with 5% bovine serum albumin (BSA). For IHC, primary antibodies included mouse anti-CD3 (GB12014, Servicebio), mouse anti-CD8 (GB15068, Servicebio), rabbit anti-cleaved caspase-3 (9603S, Cell Signaling Technology), human anti-ULBP-1 (17715-1-AP, Proteintech), and human anti-ULBP-2/5/6 (AF1298, R&D Systems), applied overnight at 4 °C. Sections were incubated with HRP-conjugated secondary antibodies, developed with DAB substrate, and counterstained with hematoxylin. For immunofluorescence, mouse anti-EGFP (GB12602, Servicebio) and Alexa Fluor 488-conjugated secondary antibodies were used, with nuclei stained by DAPI. Slides were visualized using a Leica DM6 B microscope (IHC) or Olympus FV3000 confocal system (IF), and analyzed with CaseViewer software for quantification of T cell infiltration (CD3⁺/CD8⁺), apoptosis (cleaved caspase-3⁺), and ULBP expression.

**Transcriptomic Profiling**
To capture tumor-exposed macrophage transcriptomes, 5×10⁶ iBMDM-derived GFP-M or CAR-M were co-cultured with 5×10⁵ Hepa1-6 tumor cells in 10 cm dishes for 24 hours. Post-co-culture, cells were harvested via centrifugation (350 × *g* , 5 minutes, 4 °C), washed with PBS, and resuspended in FACS buffer (PBS + 2% FBS). FITC-positive macrophages were isolated using fluorescence-activated cell sorting (FACS Aria III, BD Biosciences) to exclude tumor cells. RNA was extracted from sorted populations using TRIzol reagent (Invitrogen) and quantified via NanoDrop ND-1000 (Thermo Fisher Scientific). High-quality RNA (RIN >8.0) was submitted to Tsingke Biotech (Beijing) for library preparation and sequencing on an Illumina NovaSeq 6000 platform (paired-end 150 bp reads). Raw reads were aligned to the mouse reference genome (GRCm39) using STAR v2.7.10a, and differential gene expression analysis was performed with DESeq2 (adjusted *p* < 0.05, |log₂FC| ≥1). Functional enrichment (GO/KEGG) and pathway activation (GSEA) were analyzed to identify CAR-M-specific immune regulatory networks.

**Animal Studies**

All animal procedures were approved by the Animal Use and Care Committee of the Air Force Medical University (AFMU) and performed in accordance with institutional ethical guidelines. Female C57BL/6 and BALB/c mice (6-8 weeks old) were supplied by the AFMU Experimental Animal Center and maintained under specific pathogen-free (SPF) conditions with controlled temperature, humidity, and light/dark cycles.

**Subcutaneous Tumor Model**

Hepa1-6 murine hepatocellular carcinoma cells were washed twice with PBS and resuspended at 4×10⁶ cells per 100 µL PBS. The cell suspension was injected subcutaneously into the right flank of C57BL/6 mice. On day 7 post-inoculation, mice received tail vein injections of PBS, GFP-M (2×10⁷ cells/mouse), or CAR-M (2×10⁷ cells/mouse). Tumor dimensions were measured every 3 days using a caliper, and tumor volume was calculated as V = W² × L / 2, where W represents the shortest diameter and L the longest diameter. Survival was monitored until humane endpoints were reached. For bioluminescence imaging, mice were anesthetized with isoflurane and administered D-luciferin (150 mg/kg, GoldBio, LUCK-1G) intraperitoneally. Imaging was performed 10-15 minutes post-injection using an IVIS Spectrum system (PerkinElmer), with exposure times optimized to prevent signal saturation.

**Orthotopic Transplantation Model**

For orthotopic Hepa1-6 tumor induction, donor C57BL/6 mice were first implanted subcutaneously with 4×10⁶ cells. On day 14, 8 mm³ tumor fragments were harvested and surgically engrafted into the livers of syngeneic recipients. In a parallel model, BALB/c mice underwent hepatic subcapsular injection of H22-Rae-1β cells (1×10⁶ cells/mouse). On day 7 post-implantation, C57BL/6 mice received tail vein injections of PBS, GFP-iBMDM (2×10⁷ cells/mouse), or CAR-iBMDM (2×10⁷ cells/mouse), while BALB/c mice were treated with PBS, GFP-J774A.1 (5×10⁶ cells/mouse), or CAR-J774A.1 (5×10⁶ cells/mouse) via the same route. Body weight and survival were recorded every 3 days, and tumor progression was assessed weekly by bioluminescence imaging as described above.

**Peritoneal Dissemination Model**

BALB/c mice were injected intraperitoneally with H22 or H22-Rae-1β cells (1×10⁵ cells in 200 µL PBS). On day 7, mice received intraperitoneal administration of GFP-J774A.1 (1×10⁶ cells/mouse) or CAR-J774A.1 (1×10⁶ cells/mouse). Ascites development, body weight, and survival were monitored every 3 days. Tumor burden was evaluated weekly via bioluminescence imaging following intraperitoneal D-luciferin injection. At the experimental endpoint, livers and peritoneal tissues were collected for histological examination by H&E staining and immunohistochemical analysis.

**Pulmonary Metastasis Model**

C57BL/6 mice were intravenously injected via the lateral tail vein with either Hepa1-6-luciferase cells (4×10⁶ cells in 200 µL PBS) or B16F10-Rae-1β melanoma cells (2×10⁵ cells in 200 µL PBS). Starting on day 7, mice inoculated with Hepa1-6 cells were treated intravenously with PBS (200 µL), GFP-M (2×10⁷ cells/mouse), or CAR-M (2×10⁷ cells/mouse). Mice challenged with B16F10-Rae-1β cells received PBS (200 µL), GFP-M (1×10⁶ cells/mouse), or CAR-M (1×10⁶ cells/mouse). Lung metastasis was monitored weekly by bioluminescence imaging, and body weight was recorded regularly. Survival was tracked until humane endpoints were reached.

**Tumor Recurrence Model**

C57BL/6 mice were subcutaneously injected with 4×10⁶ Hepa1-6 cells in the right flank. On day 7, mice received CAR-M (2×10⁷ cells/mouse) intravenously to induce tumor regression. After confirmed complete remission, mice were rechallenged by contralateral subcutaneous injection of 4×10⁶ Hepa1-6 cells. Tumor volume and survival were monitored as described above. Bioluminescence imaging was used to confirm the absence of tumor pre-rechallenge and to detect potential recurrence thereafter.

**T Cell Functional Assays**

Female C57BL/6J mice (6-8 weeks old) were housed under SPF conditions. Subcutaneous Hepa1-6 tumors were established as above, and mice were randomly assigned to GFP-M or CAR-M treatment groups (n = 3 per group). On day 14, mice received 2×10⁷ GFP-M or CAR-M via tail vein injection. Seventy-two hours later (day 17), mice were euthanized by CO₂ asphyxiation.

Spleens were mechanically dissociated and erythrocytes were lysed with ACK buffer (Gibco). CD3⁺ T cells were isolated using the EasySep™ Mouse T Cell Isolation Kit (STEMCELL Technologies, 19851A), achieving >95% purity. Tumors were excised, minced, and digested with 1 mg/mL collagenase IV (Sigma) and 0.02 mg/mL DNase I (Roche) for 30 minutes at 37°C. Tumor-infiltrating lymphocytes (TILs) were sorted on a BD FACS Aria III after staining with anti-CD3-APC (BioLegend, 100236).

For co-culture assays, T cells (6×10⁵) were incubated with Hepa1-6 target cells (3×10⁵) at an effector-to-target ratio of 2:1 in complete RPMI-1640 medium using 24-well flat-bottom plates. Cultures were maintained for 72 hours at 37°C and 5% CO₂ without exogenous cytokines. T cell activation was assessed by CD69 expression via flow cytometry, IL-2 and IFN-γ secretion by ELISA, and proliferation by CFSE dilution.

**CD8⁺ T Cell Depletion**

B16-Rae-1β cells (3×10⁵ in 100 µL PBS) were implanted subcutaneously into the right flank of C57BL/6 mice. On day 7, mice were randomized into three groups: PBS control, CAR-M (3×10⁶ cells/mouse), and CAR-M plus anti-CD8 depleting antibody (200 µg/mouse, administered intraperitoneally twice weekly). Tumor volume, body weight, and survival were monitored regularly. Tumor burden was assessed weekly by bioluminescence imaging.

**Combination Therapy with Anti-PD-L1**

Hepa1-6 cells (4×10⁶ in 100 µL PBS) were subcutaneously implanted into C57BL/6 mice. On day 7, mice were randomized into four groups: PBS, GFP-M (2×10⁷ cells/mouse), CAR-M (2×10⁷ cells/mouse), and CAR-M plus anti-PD-L1 (200 µg/mouse, intraperitoneally twice weekly). Tumor growth, body weight, and survival were recorded as above. Bioluminescence imaging was performed weekly to evaluate tumor burden.


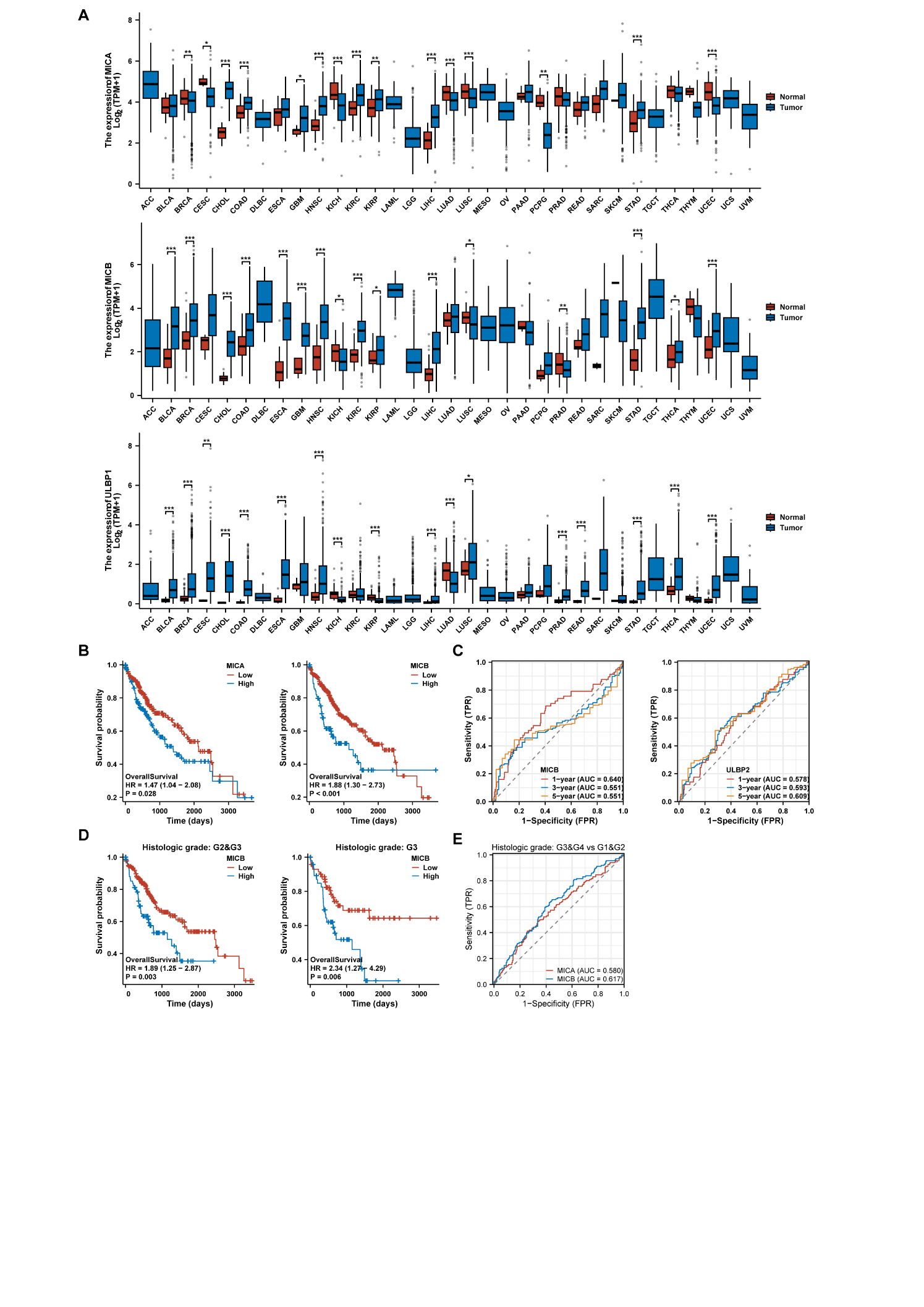
**Supplementary Figures 1 to 12**

**Fig. S1 Bioinformatics Profiling of NKG2DLs in HCC Reveals Prognostic Significance and Diagnostic Utility.** (A) Pan-cancer analysis of NKG2DL (MICA, MICB, ULBP1) expression across tumor types using TCGA datasets. (B) Kaplan-Meier survival curves demonstrating that high MICA/MICB expression correlates with reduced overall survival in HCC patients. (C) ROC curves evaluating the diagnostic accuracy of MICB/ULBP2 expression for predicting HCC progression. (D) MICB expression levels across HCC histological grades (G2, G3) and their association with patient survival. (E) ROC analysis assessing the discriminatory power of MICA/MICB expression for HCC histological grading.


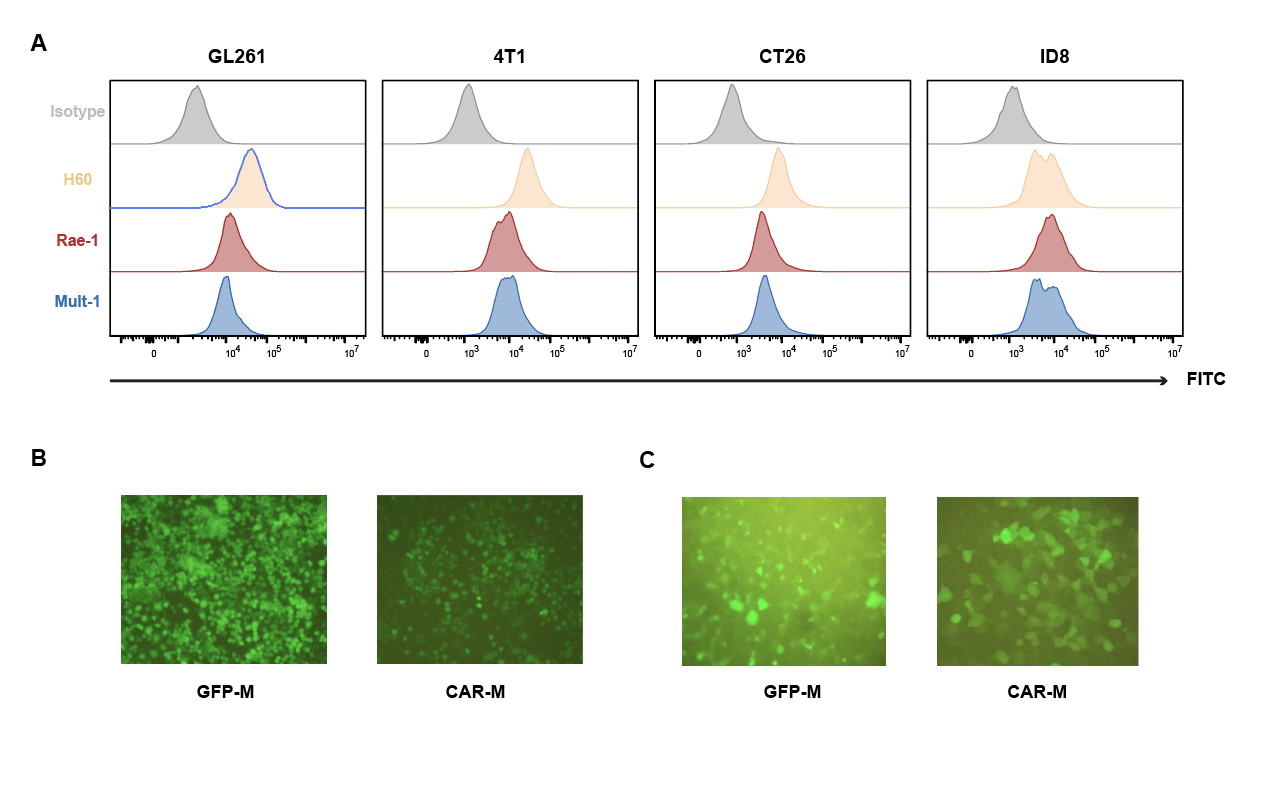


**Fig. S2 NKG2DL Expression Profiling in Murine Tumor Models and Establishment of CAR-M Stable Cell Lines.** (A) Flow cytometry analysis of NKG2D ligand (NKG2DL) expression on murine tumor cell lines (GL261, 4T1, CT26, ID8). (B-C) Generation of stable CAR-M and control macrophage lines via lentiviral transduction in iBMDM (B) and J774A.1 cells (C). EGFP expression confirmed successful transduction.


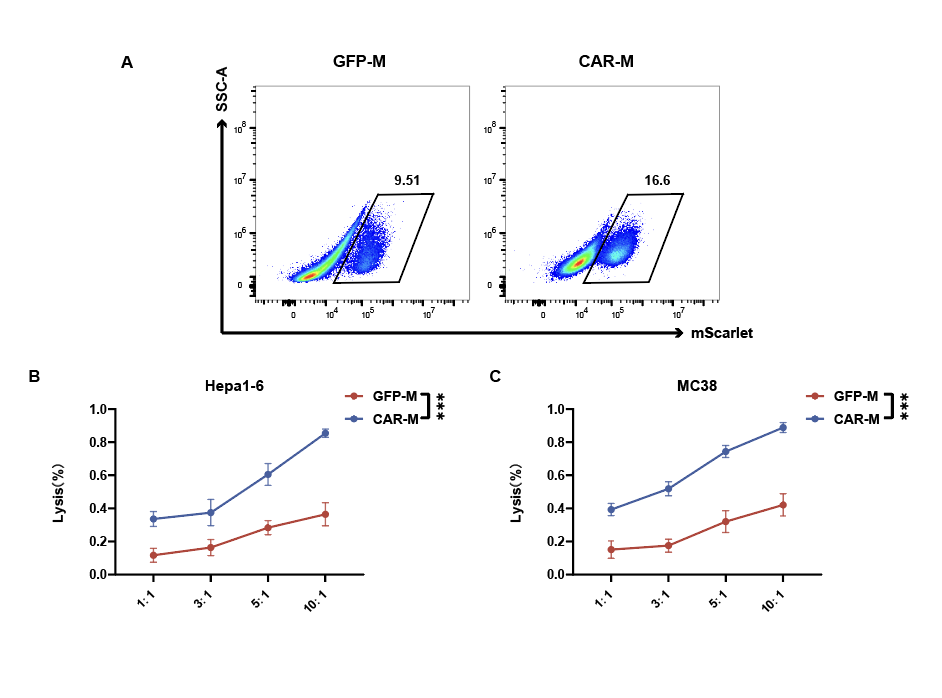


**Fig. S3 Functional validation of J774A.1 CAR macrophages *in vitro*.** (A) Flow cytometry quantification of phagocytic activity in J774A.1 CAR-M co-cultured with Hepa1-6 tumor cells at an E:T ratio of 1:1 for 1 hour. (B-C) Luciferase-based cytotoxicity assays showing dose-dependent tumor cell lysis by CAR-M after 48-hour co-culture at 10:1 E:T ratios against Hepa1-6 (B) and MC38 (C) cells. Experiments were repeated thrice independently. Data represent mean ± SD of triplicate technical replicates. Statistical significance was determined by one-way ANOVA with Tukey’s correction (*P < 0.05, **P < 0.01, ***P < 0.001).


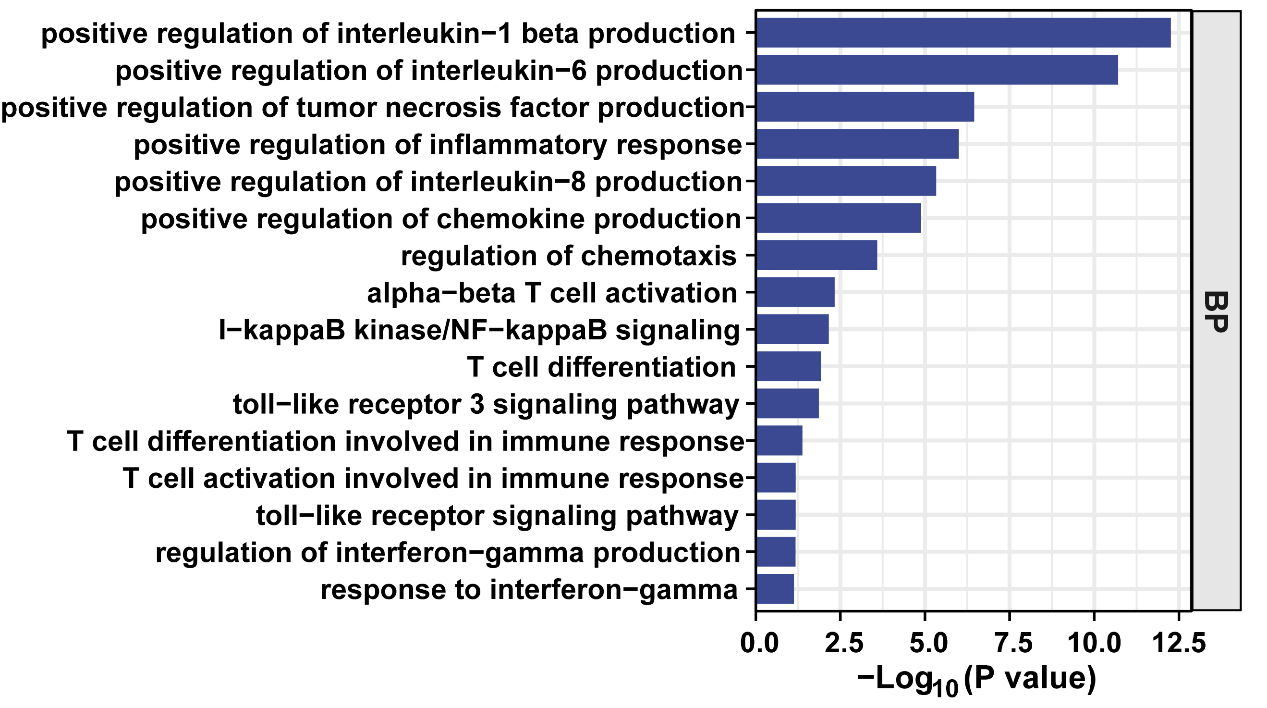


**Fig. S4 Transcriptional Profiling Reveals CAR-M Enrichment of Pro-Inflammatory Signaling Pathways.** Gene Ontology Biological Process (GO BP) analysis of RNA-Seq data from GFP-M and CAR-M co-cultured with Hepa1-6 cells (E:T ratio 10:1, 24 hours). CAR-M exhibited significant enrichment in pro-inflammatory pathways (e.g., cytokine signaling, NF-κB activation, antigen processing) compared to GFP-M controls. Three biological replicates per group validated pathway activation.


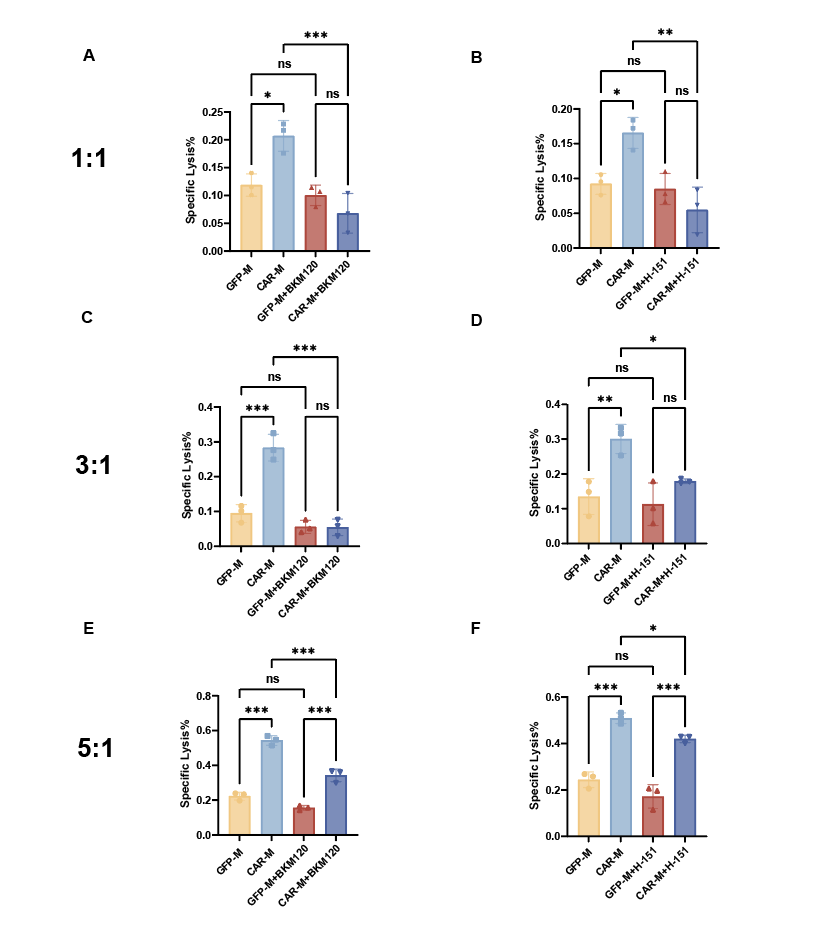


**Fig. S5 Functional roles of PI3K-AKT and cGAS-STING signaling in CAR-M‑mediated cytotoxicity.** Luciferase-based cytotoxicity assays were performed to assess the effect of pathway inhibition on CAR-M‑induced killing of Hepa1-6 cells.

(A, C, E) CAR-M cytotoxicity was suppressed by the PI3K inhibitor BKM120 at effector-to-target (E:T) ratios of 1:1, 3:1, and 5:1. (B, D, F) Similarly, the STING inhibitor H-151 impaired CAR-M‑mediated tumor cell lysis at the corresponding E:T ratios. Data are representative of three independent experiments and shown as mean ± SD of technical replicates (n=3). Statistical analysis was performed using one-way ANOVA with Tukey’s post hoc test (*P < 0.05, **P < 0.01, ***P < 0.001).


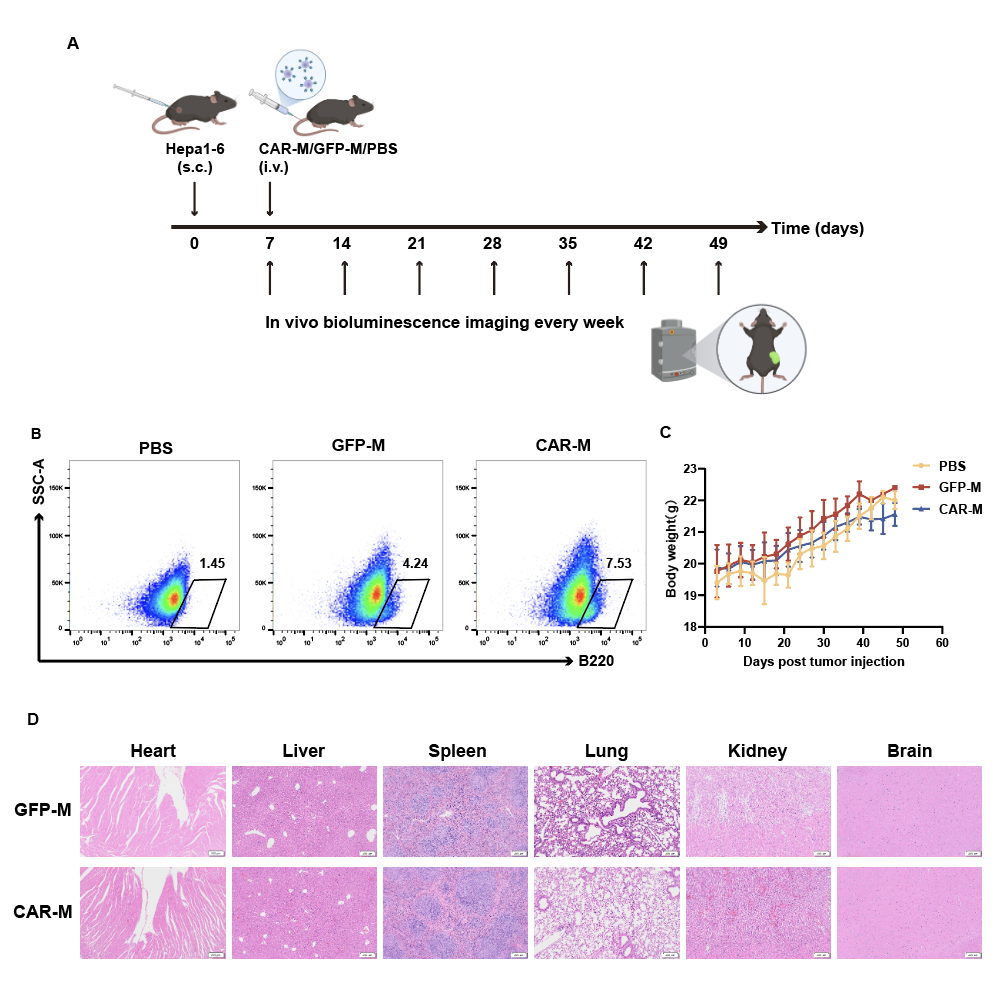


**Fig. S6 CAR-M treatment promotes B cell recruitment into tumors and demonstrates systemic safety.** (A) Experimental timeline for subcutaneous Hepa1-6 tumor establishment in C57BL/6 mice and subsequent treatment with PBS, GFP-M, or CAR-M via tail vein injection on day 7. (B) Flow cytometric analysis of tumor-infiltrating immune cells harvested 5 days post-treatment. CAR-M administration significantly increased the proportion of B lymphocytes (CD45⁺CD3⁻B220⁺) within tumors compared to control groups. (C) Body weight changes in mice throughout the treatment period. (D) Representative H&E-stained sections of major organs (heart, liver, spleen, lung, kidney, and brain) collected from mice in the GFP-M and CAR-M treatment groups. Scale bar: 200 μm.


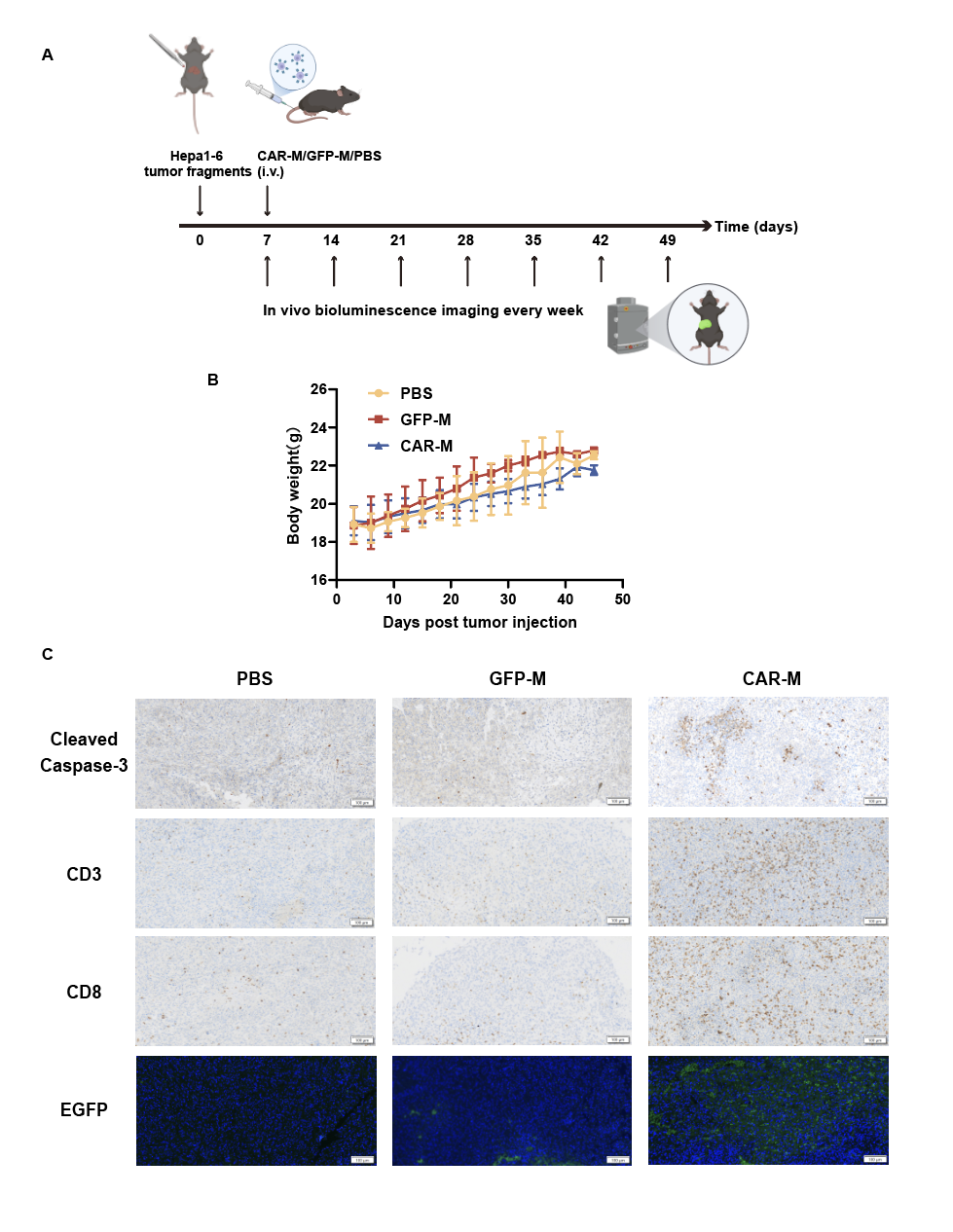


**Fig. S7 CAR-M infiltrates orthotopic liver tumors and promotes T cell activation in situ.** (A) Experimental timeline: C57BL/6 mice bearing orthotopic Hepa1-6 tumors received tail vein injections of PBS, GFP-M, or CAR-M on day 7 post-implantation. Tissues were collected for analysis 5 days post-treatment. (B) Longitudinal body weight changes of mice throughout the experimental period. (C) IHC and IF analysis of liver tumor sections. IHC staining detected CD3⁺ and CD8⁺ T cell infiltration, along with cleaved caspase-3⁺ apoptotic cells. EGFP signal was used to visualize adoptively transferred macrophages, indicating CAR-M localization within tumor regions.

**
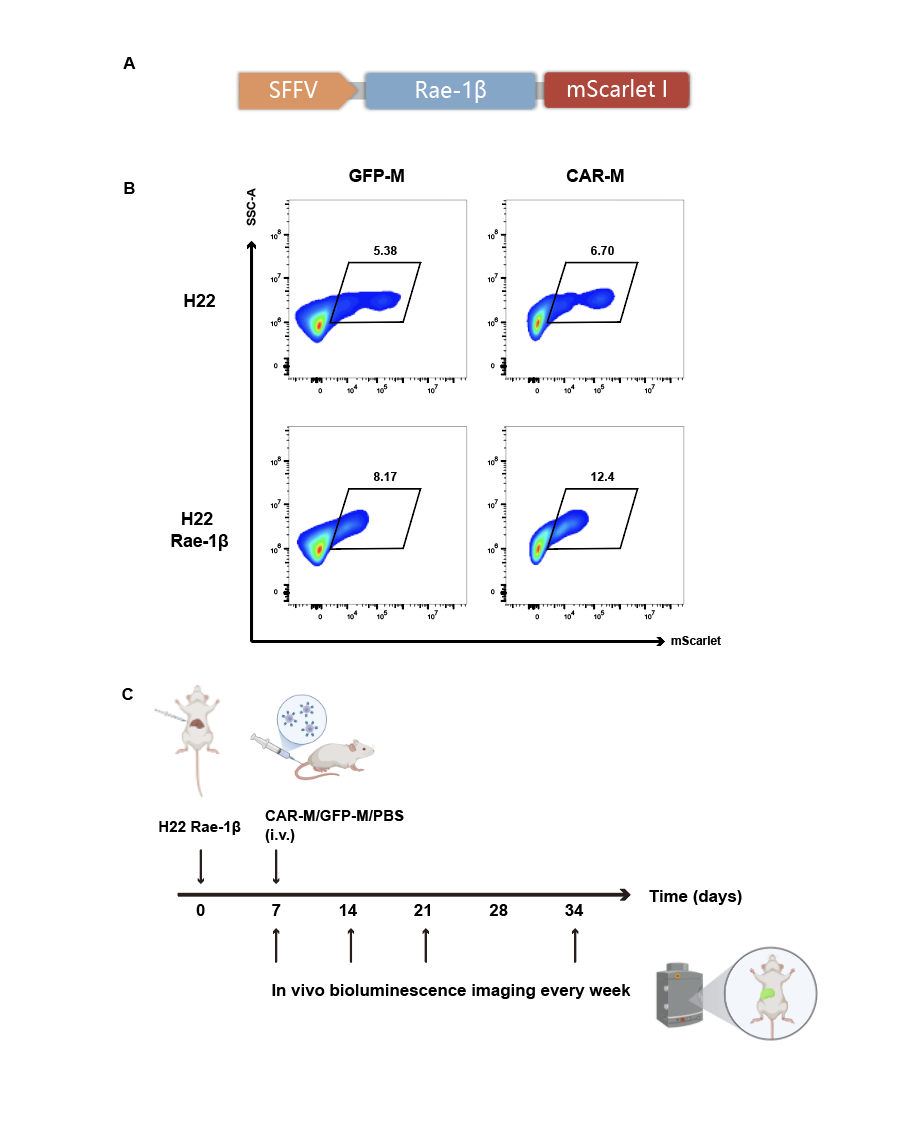
**

**Fig. S8 CAR-M mediates specific targeting of Rae-1β-overexpressing tumors through enhanced phagocytosis.** (A) Schematic of the lentiviral construct used for stable expression of a truncated Rae-1β extracellular domain fused to mScarlet and a puromycin resistance gene.(B) Phagocytic activity of GFP-M or CAR-M against H22 (NKG2DL-low) and H22-Rae-1β (NKG2DL-high) cells, quantified by flow cytometry after 1 h of co-culture at an E:T ratio of 1:1. CAR-Ms exhibited significantly enhanced phagocytosis of H22-Rae-1β targets. Data are from three independent experiments (**P < 0.01, ***P < 0.001).(C) Experimental scheme of the orthotopic H22-Rae-1β liver tumor model in BALB/c mice, used for cross-strain validation of CAR-M therapeutic efficacy.


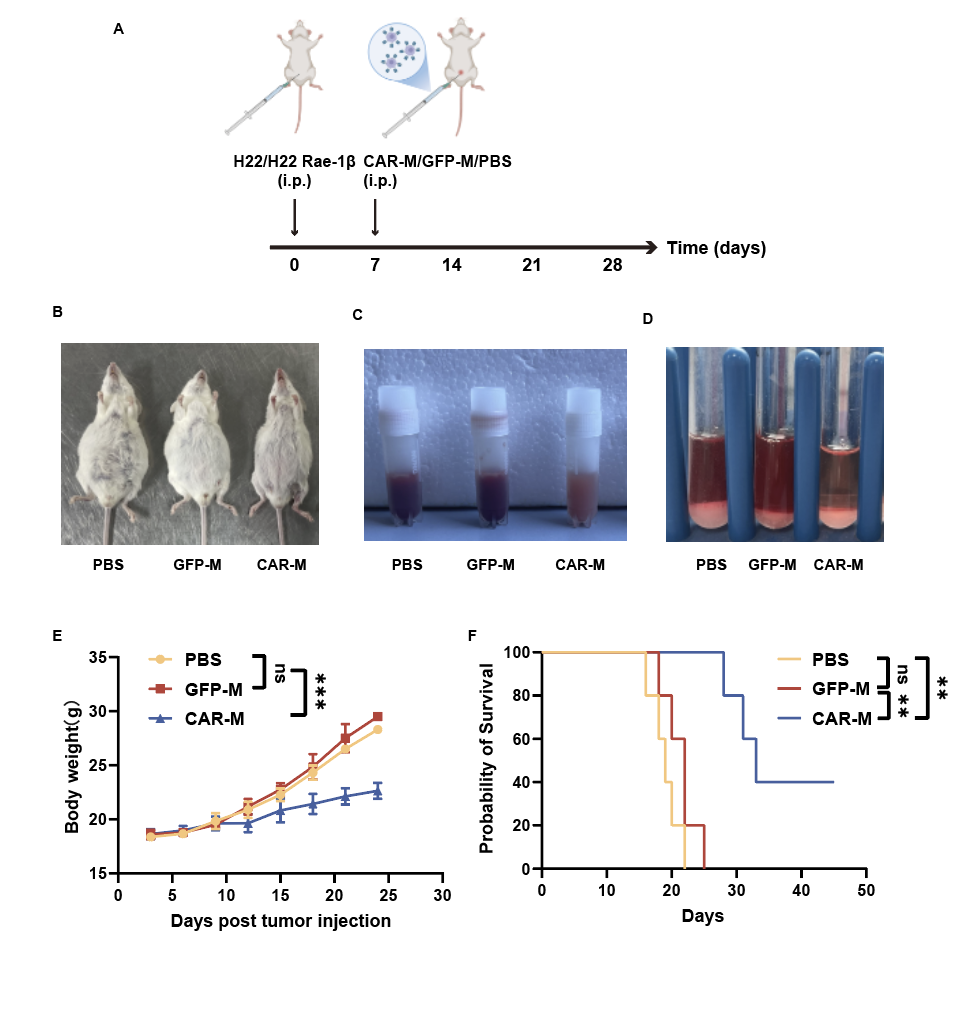


**Fig. S9 CAR-M treatment inhibits peritoneal dissemination of H22 hepatocellular carcinoma.** (A) Experimental timeline: BALB/c mice were intraperitoneally inoculated with H22 cells and treated on day 7 with PBS, GFP-J774A.1, or CAR-J774A.1 (1×10⁶ cells per mouse). (B) Representative images and quantification of ascites volume collected two weeks post-treatment, showing that CAR-M administration significantly reduced malignant ascites accumulation and hemorrhagic presentation. (C, D) Gross appearance (C) and supernatant clarity after RBC lysis (D) of ascitic fluid, reflecting reduced intraperitoneal tumor burden in the CAR-M group. (E) Body weight changes monitored weekly; CAR-M treatment ameliorated tumor-associated cachexia (n = 5 mice per group). (F) Kaplan-Meier survival analysis demonstrated that CAR-M significantly prolonged overall survival.

**
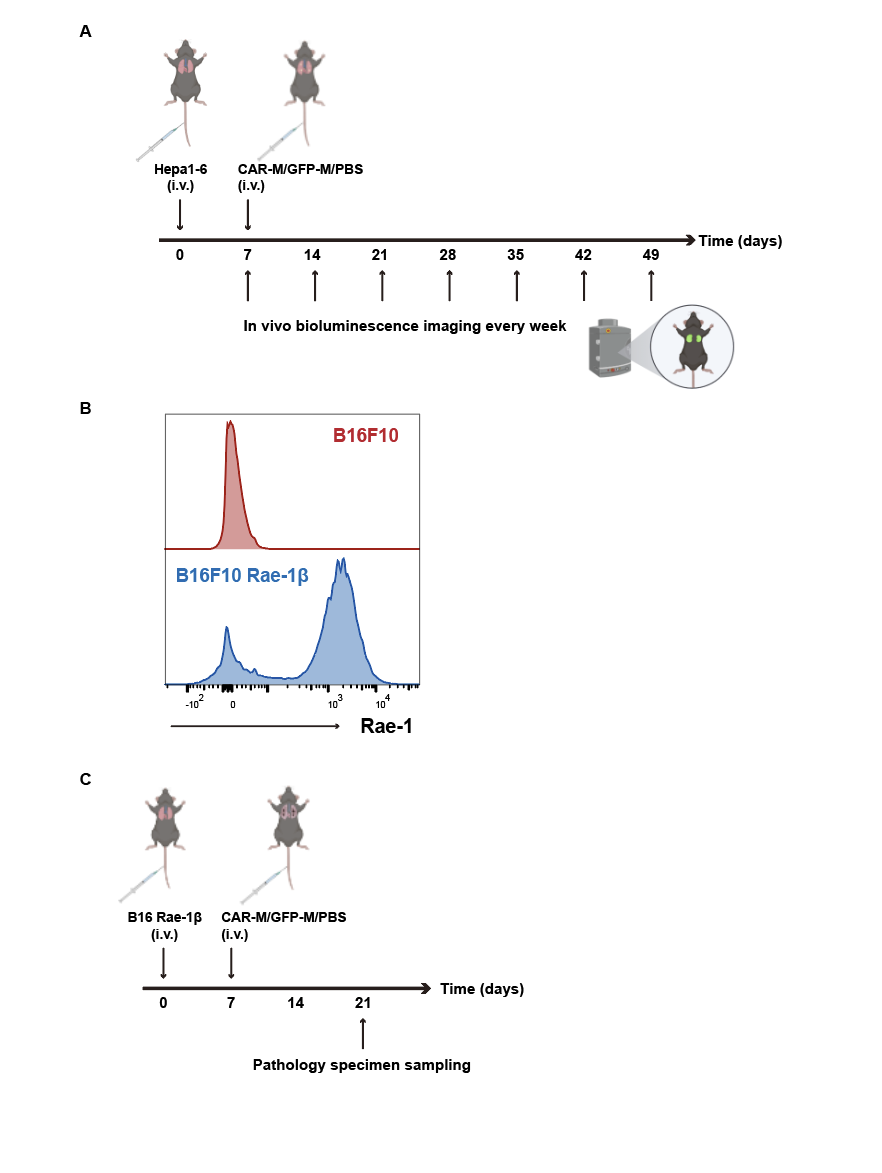
**

**Fig. S10 Experimental schematics for pulmonary metastasis models and validation of Rae-1β expression.** (A) Treatment timeline for the Hepa1-6 pulmonary metastasis model in C57BL/6 mice. (B) Flow cytometric analysis confirming stable overexpression of Rae-1β in B16F10-Rae-1β cells compared to parental B16F10 controls. (C) Treatment timeline for the B16F10-Rae-1β pulmonary metastasis model in C57BL/6 mice.

**
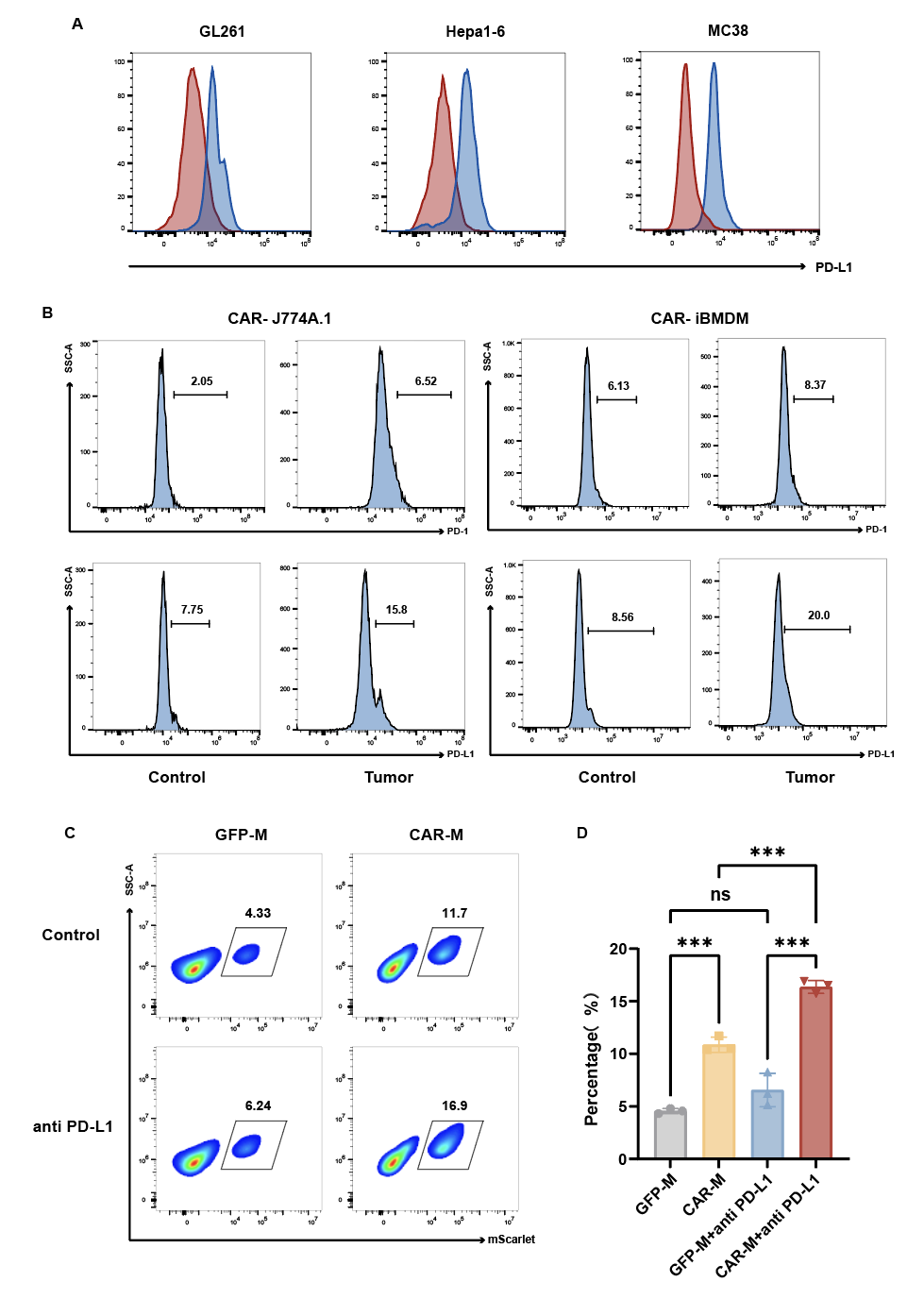
** **Fig. S11 PD-L1 blockade potentiates the phagocytic function of CAR-M *in vitro*.** (A) Flow cytometric analysis of basal PD-L1 expression on the surface of murine tumor cell lines (GL261, Hepa1-6, MC38). (B) Expression of PD-1 and PD-L1 on iBMDM and J774A.1 macrophages under basal conditions or after exposure to HER2-positive tumor cells. (C, D) Phagocytic capacity of GFP-M or CAR-M against Hepa1-6 cells at a 1:1 E:T ratio, in the presence or absence of an anti-PD-L1 antibody. (C) Representative flow cytometry plots; (D) Quantification of phagocytosis, demonstrating that PD-L1 blockade significantly enhances CAR-M-mediated tumor cell engulfment (***P < 0.001). Data are from three independent experiments and presented as mean ± SD of technical replicates.


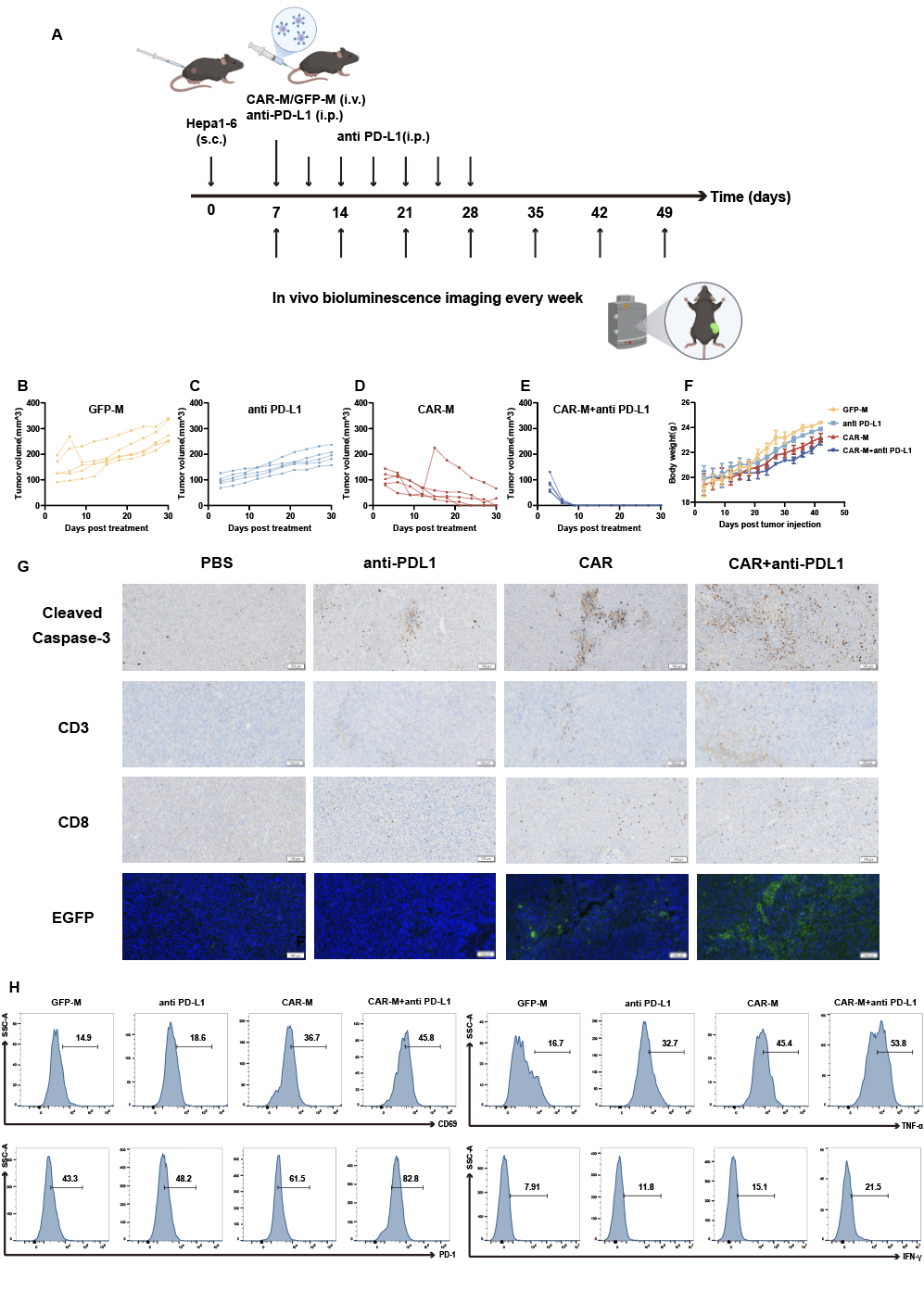


**Fig. S12. Combined CAR-M and anti-PD-L1 therapy enhances intratumoral T cell infiltration and activation.** (A) Experimental timeline for subcutaneous Hepa1-6 tumor models. Mice were treated with PBS, anti-PD-L1 (200 μg, i.p., twice weekly), CAR-M (2×10⁷ cells), or the combination of CAR-M and anti-PD-L1. (B-E) Individual tumor growth curves in mice treated with GFP-M (B), anti-PD-L1 (C), CAR-M (D), or CAR-M plus anti-PD-L1 (E). (F) Body weight changes monitored throughout the treatment period. (G) Representative IHC images of tumor sections collected 5 days post-treatment, showing CD8⁺ T cell infiltration, EGFP⁺ CAR-M localization, and cleaved caspase‑3⁺ apoptotic cells. (H) Flow cytometric analysis of tumor-infiltrating T cell subsets, including activated (CD3⁺CD69⁺), cytokine-producing (CD3⁺TNF‑α⁺/IFN‑γ⁺), and exhausted (CD3⁺PD‑1⁺) populations. Data are representative of two independent experiments. *P < 0.05, **P < 0.01, ***P < 0.001.
